# Effect of Spatial Distribution, Storage, and Cooking Methods on the Volatile Profile of Atlantic Salmon (*Salmo salar*): Influence of Pre-Harvest Rearing Conditions, Sex and Presence of Skin

**DOI:** 10.3390/foods15122124

**Published:** 2026-06-12

**Authors:** Manpreet Kaur, Md Zakir Hossain, Kevin J. Fisher, Sheryl Barringer

**Affiliations:** 1 Department of Food Science and Technology, The Ohio State University, Columbus, OH 43210, USA; kaur.333@osu.edu (M.K.); hossain.154@osu.edu (M.Z.H.); 2 School of Environment and Natural Resources, The Ohio State University, Columbus, OH 43210, USA; fisher.645@osu.edu

**Keywords:** Atlantic salmon, spatial variability, storage conditions, cooking methods, lipid oxidation, fish muscle, off-odor formation, flavor stability, SIFT-MS

## Abstract

Off-odor volatiles limit the acceptability of Atlantic salmon. This study investigated the effects of spatial distribution within the fillet, storage conditions, and cooking methods on the volatile profile of salmon and evaluated how pre-harvest rearing conditions, sex, and the presence of skin influence volatile compound formation during storage and cooking. Volatiles were classified as lipid-derived, protein-derived, and environmental contaminants. Spatial distribution within the fillet influenced volatile formation, with the head region exhibiting higher concentrations than the center and tail, reflecting differences in lipid distribution and precursor availability. During storage, fillets stored on ice generally exhibited higher volatile concentrations than samples frozen immediately, particularly for lipid-derived and environmental compounds, consistent with continued biochemical and microbial activity during chilled holding, whereas frozen storage preserved the biochemical state of the fillet. The magnitude of these differences depended on pre-harvest rearing conditions, the presence of skin, and harvest age. Cooking significantly increased volatile concentrations compared to raw fillets, with dry-heat methods, particularly baking, producing the highest levels, while boiling resulted in lower concentrations due to leaching into the cooking medium. Lower volatile formation was generally associated with cool-reared fish, male fillets, and muscle-only samples, while warm-reared, female, and skin-on samples exhibited greater volatile formation or retention, reflecting differences in precursor availability and tissue structure. These findings demonstrate that volatile formation in salmon is governed by the interaction between precursor accumulation during growth, spatial variability within the fillet, and transformation during post-harvest storage and cooking.

## 1. Introduction

Flavor is a critical determinant of consumer acceptance of fish products, with the concentration of volatile compounds playing a central role in defining aroma characteristics. In salmon, volatile compounds originate from multiple pathways, including lipid oxidation [1,2,3], protein degradation [4,5,6,7], and environmental contaminants [8,9,10,11,12] from precursors accumulated during growth. These compounds contribute to the overall sensory profile of salmon and are influenced by both tissue composition [13] and the conditions to which the fish is subjected during processing [13,14].

The volatile profile of salmon is shaped by a combination of pre-harvest and post-harvest factors [13,14]. Pre-harvest conditions, such as rearing temperature, light regime, and biological factors including sex and growth stage, influence tissue composition, particularly lipid content and fatty acid distribution, as well as the accumulation of environmentally derived compounds [13,14]. These factors determine the availability and distribution of precursors within the fillet [15]. Spatial variability within the fillet represents an additional factor influencing volatile profiles. Lipid distribution and tissue composition are not uniform along the fillet, with differences between regions such as the head, center, and tail [15]. This heterogeneity can influence precursor availability and subsequent volatile compound formation, leading to variation in measured volatile profiles depending on sampling location.

Post-harvest processes, including cooking and storage, influence volatile profiles by governing the transformation, release, and retention of these precursor compounds through thermal, enzymatic, and physical mechanisms [16,17,18]. Storage conditions also contribute to changes in volatile profiles [19,20,21]. Chilled storage allows continued biochemical and microbial activity [20,21,22], whereas frozen storage slows or halts these processes [19,21,22]. These differences can affect both the generation of new volatile compounds and the release of existing compounds from the tissue matrix [21,23,24]. Cooking is a major processing step that alters the chemical and physical structure of fish tissue [16,25]. Thermal processing promotes reactions such as lipid oxidation and protein degradation, while also affecting moisture content, oxygen exposure, and mass transfer processes [25,26]. Different cooking methods vary in temperature profiles, heat transfer mechanisms, and interaction with the surrounding medium, which can influence the formation and retention of volatile compounds in fish fillets [25,26].

While cooking and storage are well recognized for their effects on flavor and volatile compound formation in fish, pre-harvest conditions, presence or absence of skin, and spatial variability within the fillet have largely been studied from an aquacultural and nutritional perspective. The interaction between these factors and post-harvest processes, particularly their influence on volatile compound formation during cooking and storage, remains less explored. Our previous work has established how rearing, physiological, and processing factors influence how volatiles evolve over time during refrigerated storage [13,15]. However, these studies do not address how variability within the fillet, ice versus frozen storage, and cooking, collectively influence volatile formation. Therefore, the objective of this study was to systematically investigate the effects of spatial variability within the fillet, storage conditions, and cooking methods on volatile compound formation in salmon and to evaluate how pre-harvest conditions, biological factors, and the presence of skin influence off-odor volatile compound formation during storage and cooking.

## 2. Materials and Methods

### 2.1. Experimental Design and Rearing Conditions

All animal handling and experimental procedures were approved by The Ohio State University Institutional Animal Care and Use Committee (IACUC protocol numbers 2008A0220-R5 and 2008A021-R5; approval date: 14 April 2024). Atlantic salmon (*Salmo salar*) were reared at the Aquaculture Laboratory, School of Environment and Natural Resources, The Ohio State University, using a recirculating aquaculture system (RAS) designed to allow controlled manipulation of water temperature and photoperiod. The RAS consisted of multiple 400 L tanks connected to a mechanical filtration unit and sump pump system. Water flow could be switched between recirculated chilled water and fresh municipal water using cutoff valves, allowing independent environmental control between tank groups. The rearing conditions for this study have been described in detail previously [14]. Briefly, fish were reared under two rearing conditions, and the fish analyzed in the present study are from the second and third harvests [14]. Fish were fed extruded salmon feed (Skretting, UT, USA) composed of 48% crude protein and 18% crude lipid ad libitum throughout the study.

### 2.2. Harvest and Post-Harvest Fillet Processing

At each harvest point, six salmon were randomly selected, consisting of three fish reared under cool conditions (13.1 ± 0.85 °C with 12 h light–12 h dark cycle) and three fish reared under warm conditions (20.3 ± 1.95 °C with continuous light). Fish were mechanically stunned by a percussive blow to the head, followed by immediate bleeding via dorsal artery severance. Each fish was filleted and processed into two sample types: muscle-only (without skin) and muscle with skin attached. For muscle-only, the skin was removed manually to ensure complete separation from the underlying muscle tissue. Both sample types were used for subsequent analysis; in muscle-only samples, subcutaneous fat and red muscle were largely excluded.

The experimental design consisted of three main components. First, spatial distribution within the fillet was evaluated by comparing volatile profiles among the head, center, and tail regions. Second, the effect of storage was evaluated by comparing ice-stored and frozen samples from muscle-only and skin-on fillets across pre-harvest rearing conditions and harvest ages. Third, the effect of cooking was evaluated by comparing raw, baked, pan-fried, and boiled samples while considering pre-harvest rearing conditions, sex, and the presence or absence of skin.

#### 2.2.1. Spatial Distribution Sample Preparation

Each fillet (muscle without skin from the third sampling) [14] was divided longitudinally into three equal regions: the anterior portion nearest the head, the middle portion of the fillet, and the posterior portion nearest the tail. These regions are referred to as the head, center, and tail regions, respectively. To minimize within-region variability, muscle tissue from each section was minced and thoroughly mixed, and representative 2 g samples were taken from each region for volatile analysis. Samples were stored at −80 °C prior to analysis and thawed at room temperature for 30 min before analysis.

#### 2.2.2. Storage Sample Preparation

Ice and frozen storage conditions were applied to evaluate their effects on volatile compound formation in fillets from the second and third sampling. Samples were excised from the same location on the head side of each fillet to ensure consistency across treatments. For ice storage, samples (2 g) were immediately placed into food-grade polyethylene zip-lock bags. Samples were then stored on ice at 0 ± 2 °C for 2–3 h prior to volatile analysis. For frozen storage, prepared samples (2 g) were placed into food-grade polyethylene zip-lock bags and stored at −80 °C for 90 days prior to analysis. Following frozen storage, muscle samples (skin-on and skin-off) were removed from the freezer and thawed at room temperature (22 ± 2 °C) for 30 min before volatile analysis. The same thawing procedure was applied to all frozen samples to standardize sample temperature and minimize handling-related variability before headspace equilibration.

#### 2.2.3. Cooking Sample Preparation

Salmon fillets from the second sampling were prepared for cooking using standardized sampling procedures. For all cooking treatments, samples (2 g) were excised from the same location on the head side of each fillet to ensure consistency across treatments. Samples were subjected to three different cooking methods: baking, pan-frying, and boiling. For baking, samples were placed in a preheated hot air oven at 200 °C and cooked for 15 min. For boiling, samples were submerged in water maintained at 100 ± 1 °C for 15 min. For pan-frying, samples were cooked on a preheated surface at 200 °C. All cooking treatments were conducted under controlled conditions, and samples were allowed to cool to room temperature prior to volatile analysis.

### 2.3. Headspace Volatile Analysis by SIFT-MS

A 2 g portion of minced tissue was transferred to a 500 mL Pyrex bottle. To facilitate volatile release, 20 mL of 0.5% (*v*/*v*) ethanol was added, and bottles were sealed with open-top, septum-lined caps. Samples were homogenized using a vortex mixer for 1 min and equilibrated in a water bath at 42 °C for 30 min. Headspace volatile compounds were analyzed using selected-ion flow-tube mass spectrometry (SIFT-MS; Voice200ultra, Syft Technologies, Christchurch, New Zealand). Analyses were performed in selected-ion monitoring mode using H_3_O^+^, NO^+^, and O_2_^+^ precursor ions. Volatile compounds quantified in this study are the same as in our previous study [14]. Compound concentrations were calculated using established ion–molecule reaction rate coefficients. Instrument calibration was verified using a certified gas standard containing benzene, ethylbenzene, toluene, and xylene isomers prior to sample analysis. Headspace sampling was conducted using a 14-gauge passivated needle, with the inlet temperature maintained at 175 °C. Each sample was analyzed over a 120 s acquisition period. Three analytical replicates were performed per sample type. An empty Pyrex bottle was analyzed as an instrument/background blank, and a procedural blank containing 20 mL of 0.5% ethanol was prepared and equilibrated under the same conditions as the samples to account for reagent- and material-derived background volatiles.

### 2.4. Statistical Analysis

Statistical analyses were conducted using JMP^®^ Pro Version 16.0.0 (SAS Institute Inc., Cary, NC, USA). Figures were generated using MATLAB^®^ R2024b Update 5 (MathWorks, Natick, MA, USA). For heatmap visualization, volatile concentration data were uploaded to MetaboAnalyst. Data were log-transformed and normalized by compound prior to hierarchical clustering. Clustering was performed using the default MetaboAnalyst settings. Volatile compounds were analyzed using analysis of variance (ANOVA) appropriate to each experimental design. Spatial differences within the fillet were evaluated using a mixed-effects model with the fillet region as a fixed effect and fish as a random effect. Storage data were analyzed using multi-factor ANOVA with storage condition, pre-harvest rearing conditions, presence of skin, harvest age, and their interaction terms as fixed effects. Cooking data were analyzed using multi-factor ANOVA with the cooking method, pre-harvest rearing conditions, sex, tissue type, and their interaction terms as fixed effects. These models were used to compare the relative influence of processing, biological, and rearing factors on volatile concentrations. When significant effects were observed, Fisher’s least significant difference (LSD) test was used for post hoc comparisons. Statistical significance was defined at *p* ≤ 0.05. For each factor, three biological replicates were included, with a total of six fish analyzed per harvest.

## 3. Results

### 3.1. Effect of Spatial Distribution Within the Fillet on Off-Odor Volatiles in Raw Salmon

**Figure 1:**
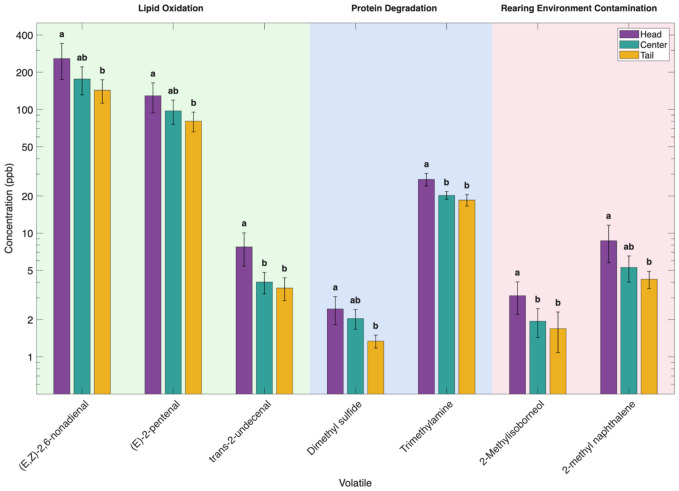
Effect of spatial distribution within the fillet on the off-odor volatile compounds. Volatile concentration is plotted on a base-10 logarithmic (log10) scale. Different letters indicate a significant difference in each volatile (*p* < 0.05).

Lipid composition varies within fillets; thus, it is important to determine how spatial distribution within a fillet influences off-odor volatile compound formation in raw fillets. Samples were taken from the head, center, and tail of raw salmon filets, and volatile concentrations were measured. Among the volatiles quantified, 41 compounds exhibited significantly higher concentrations in the head region, followed by the center, with the lowest concentrations in the tail (Table A1). These compounds were classified as lipid oxidation volatiles, protein degradation volatiles, and environmental contamination volatiles [13] (Figure 1, Table A1). Across all categories, this head-to-tail pattern demonstrates pronounced spatial heterogeneity in volatile chemistry within the fillet.

Although all lipid oxidation volatiles tended to decrease from head to center to tail, 23 lipid oxidation volatiles showed significantly higher concentrations in the head region (Table A1). For all of these volatile compounds, the head region differed significantly from the tail, while the center region was frequently not significantly different from the head or the tail (Figure 1, Table A1). The significantly higher concentrations of these lipid oxidation volatiles in the head region indicate spatial heterogeneity in lipid-derived off-odor development along the fillet. Fatty acid composition showed a similar head-to-tail trend [15]. The head region contained significantly higher lipid content (6.08%), followed by the center region (5.06%) and the tail region (2.77%), which exhibited a significantly lower lipid content. This distribution indicates that higher lipid levels in the head region were associated with a greater formation of lipid oxidation volatiles, whereas lower lipid levels in the tail were associated with reduced volatile formation.

Most (13) of the protein degradation volatile compounds also exhibited significantly higher concentrations in the head region, followed by the center, and exhibited the lowest concentrations in the tail (Figure 1, Table A1). The significantly higher concentrations of these compounds in the head region indicate higher protein degradation in this portion of the fillet, likely because higher lipid oxidation leads to an increase in the rate of protein degradation [27].

Among the environmental contamination volatile compounds, 2-methylisoborneol (2-MIB) and 2-methylnaphthalene showed significantly higher concentrations in the head region compared to the center and tail (Figure 1, Table A1). These volatiles are lipophilic and preferentially deposit into lipid depots, resulting in greater accumulation in regions with higher lipid content and lower accumulation in regions with lower lipid content [13,14], which explains why they follow the same trend as lipid oxidation volatiles.

Lipid content influences volatile formation pathways in salmon, affecting lipid oxidation, protein degradation, and environmentally associated off-odor volatile compounds [13]. The higher concentrations of off-odor volatile compounds in the head region, as compared to the center and tail regions, highlight the importance of standardizing and reporting sampling location because differences in the fillet region can lead to a substantial variation in measured volatile profiles. The higher concentration of lipid oxidation and degradation-related volatiles in the head region also suggests that protective methods for preservation of fish fillets, such as antioxidant treatments or edible films, may benefit from region-specific design, with greater protection targeted toward the head portion of the fillet than the tail. Incorporating spatial variability into preservation approaches may therefore improve control of lipid oxidation, reduce deterioration, and increase the shelf life of fish products.

### 3.2. Effect of Storage Temperature, Pre-Harvest Rearing Conditions, and Presence of Skin on the Volatile Profile of Raw Salmon Fillets

In raw fillets, storage temperature (ice vs. frozen), pre-harvest rearing conditions, presence of skin, and harvest age influenced the volatile profile of salmon (Table A2). Storage temperature significantly influenced lipid-derived (*p* = 0.0356) and environmental-derived volatiles (*p* = 0.0039), but not protein-derived volatiles (*p* = 0.0912), while pre-harvest rearing conditions showed a consistent significant effect on all volatiles (lipid: *p* = 0.0116; protein: *p* = 0.0095; environmental: *p* = 0.0102). Storage temperature and pre-harvest rearing conditions also showed pre-harvest rearing conditions × storage temperature interaction (lipid: *p* = 0.0256; protein: *p* = 0.0403; environmental: *p* = 0.0385). The presence of skin significantly affected lipid-derived volatiles (*p* = 0.0224) and contributed through interactions to protein (storage temperature × presence of skin, *p* = 0.0053) and environmental volatiles (storage temperature × presence of skin, *p* = 0.0225). Although harvest age did not show a significant independent effect (*p* > 0.05), it contributed to significant interaction effects, particularly harvest age × storage temperature (lipid: *p* = 0.0249; protein: *p* = 0.0040) and pre-harvest rearing conditions × harvest age × storage temperature (lipid: *p* = 0.0129; protein: *p* = 0.0121; environmental: *p* = 0.0226). Lipid-derived volatiles showed a significant four-way interaction among pre-harvest rearing conditions, harvest age, storage temperature, and presence of skin (*p* = 0.0447).

#### 3.2.1. Effect of Storage Temperature (Ice Versus Frozen Storage) on the Volatile Profile of Raw Salmon Fillets

**Figure 2:**
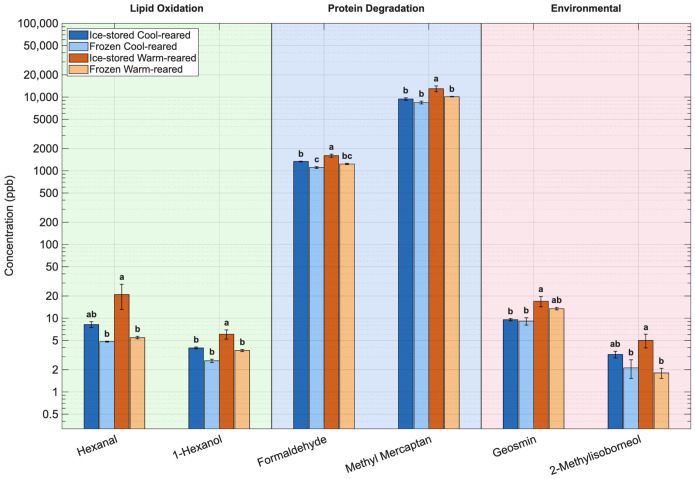
Volatile compounds in samples reared in cool and warm conditions and stored on ice or frozen, for muscle-only fillets harvested at 301 days. Volatile concentration is plotted on a base-10 logarithmic (log10) scale. Different letters indicate a significant difference in each volatile (*p* < 0.05).

Storage temperature significantly influenced the volatile profile of raw salmon fillets (Figure 2). Across all of the volatiles, samples stored on ice generally exhibited higher concentrations than frozen samples, as would be expected (*p* < 0.05) (Table A2, Figure 2). This is likely due to continued lipid oxidation and increased release of retained environmentally derived compounds from the muscle matrix into the headspace during chilled holding, whereas frozen storage limits these processes by slowing enzymatic, oxidative, and microbial activity. Although stored on ice, fillets remain enzymatically and microbially active for a short period after harvest [19]. Residual oxygen, intact lipid substrates, and endogenous oxidative enzymes allow lipid degradation to proceed, producing characteristic oxidation products [19].

Protein-derived volatiles did not show a significant effect of storage temperature (*p* = 0.0912). This suggests that the short ice-storage period was insufficient to produce substantial protein degradation prior to analysis. In fish muscle, lipid oxidation reactions generally proceed more rapidly during storage than protein degradation pathways, which often require longer enzymatic and microbial activity to generate measurable concentrations of protein-derived volatile compounds [19,24]. Consistent with this, protein-derived volatile compounds showed less change with storage conditions (Figure 2). The observed increase in lipid-derived and environmental volatiles, alongside the limited response of protein-derived volatiles, indicates that short-term storage primarily influences volatile formation through rapid lipid oxidation and partitioning mechanisms rather than extensive protein degradation.

#### 3.2.2. Influence of Pre-Harvest Rearing Conditions on the Volatile Profile of Raw Salmon Fillets

Pre-harvest rearing conditions significantly affected the volatile profile of raw salmon fillets across all volatile classes (lipid: *p* = 0.0116; protein: *p* = 0.0095; environmental: *p* = 0.0102), with additional contributions from interaction effects with the storage temperature and the presence of skin (Table A2, Figure 2). Overall, salmon reared under warm conditions exhibited higher volatile concentrations compared with those reared under cool conditions. Warm rearing increases metabolic rate, feed intake, lipid deposition, oxidative stress, and microbial activity, leading to greater accumulation of lipid oxidation substrates, protein degradation precursors, and environmental contaminants in both muscle and skin tissues [14,15].

Pre-harvest rearing conditions govern precursor accumulation during growth, while the expression of these differences depends on post-harvest conditions. During ice storage, continued lipid oxidation, enzymatic activity, and microbial metabolism allow these precursors to be converted into volatile compounds [13,14,19], resulting in higher concentrations in warm-reared fish. In contrast, frozen storage preserves the biochemical state of the tissue at harvest [19], limiting further volatile formation and reducing the expression of rearing-dependent differences.

#### 3.2.3. Influence of Fillet Preparation Type (Muscle vs. Skin-On Muscle) on the Volatile Profile of Salmon Fillets

Preparation of the fillet as muscle only or skin-on muscle also influenced the volatile profile of the salmon, with effects that depended on volatile class and storage temperature (Table A2). The presence of skin showed a significant effect for lipid-derived volatiles (*p* = 0.0224), while the effects of protein- and environmental-derived volatiles were primarily interaction-driven (storage temperature × presence of skin interactions (protein: *p* = 0.0053; environmental: *p* = 0.0225).

**Figure 3:**
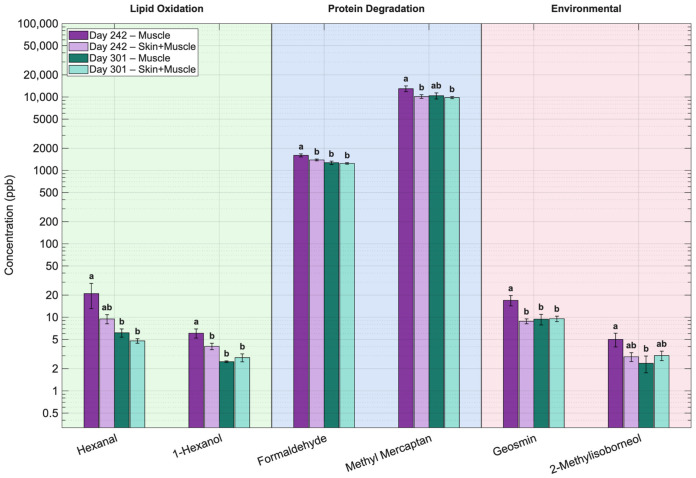
Volatile compounds in muscle and skin-on muscle samples harvested at 242 and 301 days, initially reared in warm conditions (242 days) and flipped to cool conditions (301 days), and stored frozen. Volatile concentration is plotted on a base-10 logarithmic (log10) scale. Different letters indicate a significant difference in each volatile (*p* < 0.05).

The presence or absence of skin had a minimal impact on the volatile profile under cool pre-harvest rearing conditions at both harvest times and both storage conditions (Table A2). Again, this indicates a limited precursor accumulation under cool rearing conditions. In contrast, under warm pre-harvest rearing conditions, the effect of skin was significant in frozen samples (Figure 3). This reflects the distinct effects of freezing on muscle structure and precursor release [13,15,28]. During freezing and subsequent thawing, muscle structure is disrupted by ice crystal formation, damaging adipocytes, connective tissue, and cellular membranes within the skin and adjacent muscle tissue [14,28]. This structural disruption increases tissue permeability and promotes the release of retained lipid substrates and degradation precursors during thawing [15], resulting in a greater volatile formation in skin-on frozen samples. In contrast, during ice storage, the intact skin partially limits oxygen exposure and slows the release of lipid oxidation substrates and protein degradation precursors from underlying tissues [13,15,19].

#### 3.2.4. Influence of Harvest Age on the Volatile Profile of Raw Salmon Fillets

Unlike the other factors, harvest age did not show a significant independent effect on lipid, protein, or environmental volatile concentrations (*p* > 0.05) (Table A2, Figure 3). However, significant interaction effects involving harvest age and storage temperature were observed, including harvest age × storage temperature (lipid: *p* = 0.0249; protein: *p* = 0.0040), pre-harvest rearing conditions × harvest age × storage temperature (lipid: *p* = 0.0129; protein: *p* = 0.0121; environmental: *p* = 0.0226), and pre-harvest rearing conditions × harvest age × storage temperature × presence of skin (lipid: *p* = 0.0447) indicating that the influence of harvest age depends on storage temperature, pre-harvest rearing conditions, and the presence of skin.

These results demonstrate that off-odor volatile formation in raw salmon fillets depends on the interaction between pre-harvest conditions and post-harvest storage, rather than on any single factor alone. Pre-harvest rearing conditions may be associated with oxidative stress and precursor accumulation, particularly for lipid oxidation substrates, protein degradation intermediates, and retained environmental contaminants, while storage temperature determines the extent to which lipid oxidation and environmental release occur, and protein-derived volatiles are expressed through interaction-dependent pathways. The presence of skin further modulates this response by controlling the retention and release of precursors within muscle and skin-on tissue.

### 3.3. Effect of Cooking Method on the Volatile Profile of Salmon Fillets as Influenced by Pre-Harvest Rearing Conditions, Sex, and Presence of Skin

**Figure 4:**
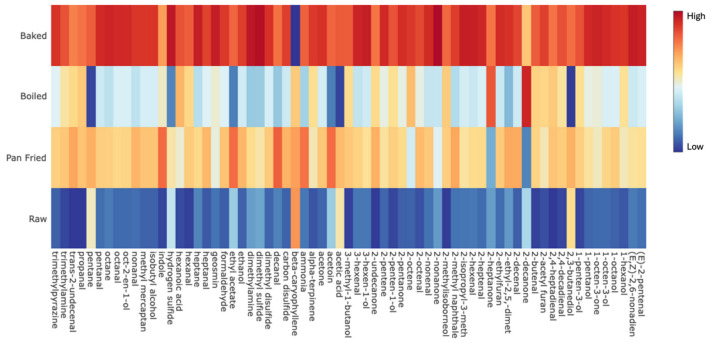
Hierarchical clustering heatmap for the effect of cooking on the volatile profile of salmon (*p* < 0.05). Red indicates a higher relative abundance, and blue indicates a lower abundance.

The cooking method significantly (*p* = 0.002) influenced the volatile profile of salmon fillets, as thermal processing promotes the formation and release of many volatiles that contribute to the aroma of cooked fish. Across treatments, many volatile compounds increased after cooking, with differences depending on the cooking method. Baked samples generally exhibited higher concentrations for many compounds, followed by pan-fried and boiled samples, while raw fillets showed the lowest concentrations for most compounds (Table A3, Figure 4). This effect on volatile concentration can be attributed to the combined effects of lipid oxidation, protein denaturation, and thermally induced release of bound precursors.

Pre-harvest factors also significantly contributed to the cooked volatile profile. Pre-harvest rearing conditions strongly influenced volatile concentrations across cooking methods (*p* < 0.001), with warm-reared fish exhibiting higher volatile levels than cool-reared fish (Table A4). This indicates that growth conditions likely influence the size and composition of the lipid and protein precursor pools entering cooking, which were subsequently transformed during thermal processing. Sex further modulated volatile formation during cooking (*p* = 0.0007), with female fillets generally exhibiting higher volatile concentrations than male fillets (Table A5), consistent with sex-dependent differences in the lipid deposition and metabolic activity in salmon [15]. The presence of skin alone did not exert an independent effect (*p* = 0.1); however, there is a significant cooking method × presence of skin interaction (*p* = 0.007) (Table A6).

#### 3.3.1. Effect of Cooking Methods on the Volatile Profile of Salmon Fillets

All cooking methods significantly increased volatile concentrations compared with raw fillets, as expected, since thermal processing is a primary driver of volatile formation in cooked salmon. Most lipid-derived volatile compounds significantly increased across all cooking methods (Table A3, Figure 4). The uniform elevation of lipid-derived aldehydes, alcohols, and acids after cooking is due to heat-accelerated lipid oxidation, particularly the thermal decomposition of lipid hydroperoxides formed from polyunsaturated fatty acids in salmon [29,30]. Aldehydes such as hexanal, heptanal, and nonanal are well-established secondary oxidation products of n-6 and n-9 fatty acids [31], while the conjugated dienals 2,4-decadienal and 2,4-heptadienal arise from further breakdown of linoleic- and arachidonic acid-derived hydroperoxides [32,33]. Protein-derived volatiles also increased significantly with cooking. Thermal processing promotes protein denaturation and degradation, leading to the formation of nitrogen- and sulfur-containing compounds such as dimethyl sulfide, dimethyl disulfide, trimethylamine, and ammonia (Table A3). These compounds arise from amino acid degradation, Strecker reactions, and a breakdown of sulfur-containing amino acids, contributing to characteristic cooked and fishy odors [34]. Environmental contaminant volatiles also showed increased concentrations after cooking (Table A3). Compounds such as geosmin and 2-methylisoborneol originate from the aquaculture environment and accumulate in lipid-rich tissues prior to harvest [13,14]. During cooking, heat disrupts tissue structure and lipid matrices, facilitating the release of these pre-existing compounds into the headspace [13,14,35].

Among cooking methods, baked samples exhibited the highest concentrations for most volatile compounds (Table A3, Figure 4). Namely, 22 lipid-derived volatiles, six protein-derived volatiles, and two environmental contaminants were significantly higher in the baked samples compared with both the fried and boiled samples (Table A3). The higher volatile concentrations observed during baking are consistent with prolonged dry-heat exposure, which promotes sustained lipid oxidation and protein degradation without a substantial physical loss of substrates or products [31]. Unlike frying or boiling, baking limits the direct removal of lipids or water-soluble compounds, allowing oxidation products to form and accumulate within the tissue matrix [29,36]. In this way, dry-heat conditions enhance the formation of aldehydes, ketones, and sulfur-containing compounds in fish [30,34].

Fried samples showed intermediate behavior, with concentrations lower than baked samples but higher than boiled samples (Table A3, Figure 4). High surface temperatures during frying accelerate lipid hydroperoxide decomposition, favoring the formation of short-chain aldehydes and hydrocarbon fragments [32,34]. However, frying also resulted in lower concentrations of many oxidation-derived volatiles compared with baking, likely due to partial lipid migration from the fillet while frying, which reduces the amount of oxidizable substrate remaining in the tissue [36,37].

Boiled samples generally exhibited the lowest concentrations for most volatile compounds (Table A3, Figure 4). The generally lower concentrations observed after boiling are due to submersion of the fillets in water, which promotes the leaching of volatile compounds and water-soluble precursors into the cooking medium [35,38]. In addition, the aqueous environment limits oxygen availability and reduces surface temperatures, thereby decreasing the extent of oxidative reactions and subsequent volatile formation [30,38].

#### 3.3.2. Influence of Pre-Harvest Rearing Conditions on the Volatile Profile of Cooked Salmon

**Figure 5:**
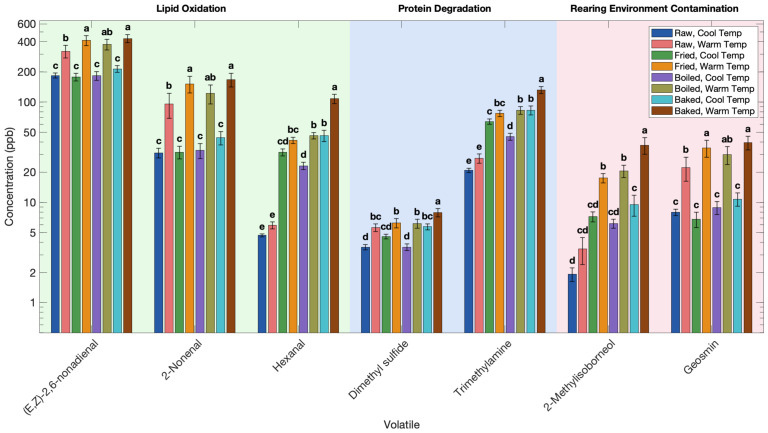
Effect of pre-harvest rearing conditions on the volatile profile of cooked salmon. Volatile concentration is plotted on a base-10 logarithmic (log10) scale. Different letters indicate a significant difference in each volatile (*p* < 0.05).

Just as pre-harvest rearing conditions significantly influenced the volatile profile of raw salmon, in cooked salmon, warm-reared fish again generally exhibited higher volatile concentrations than cool-reared fish across baked, pan-fried, and boiled treatments (Table A4, Figure 5). These differences were larger after cooking, indicating that thermal processing enhances the expression of rearing-dependent differences already present in raw fillets. Pre-harvest rearing conditions likely affect the availability of volatile precursors entering cooking, while the cooking method determines the extent to which these precursors are transformed into volatile compounds during thermal processing.

Lipid-derived compounds such as hexanal and 1-hexanol exhibited consistently higher concentrations in warm-reared fish across cooking methods (Figure 5), reflecting enhanced thermal conversion of oxidation-susceptible lipid substrates [29,30,31,32,33]. Environmental compounds such as geosmin similarly showed greater release in warm-reared fish following cooking, consistent with increased partitioning of these compounds into lipid-rich tissues prior to harvest [13,14,15].

As established in raw fillets (Section 3.2), warm rearing increased precursor availability prior to cooking. Thermal processing subsequently transformed these precursors through heat-driven lipid oxidation, protein degradation, and release of retained environmental compounds, resulting in greater volatile formation in warm-reared fish. Baking produced the strongest expression of rearing-dependent differences, consistent with prolonged dry-heat exposure promoting extensive thermal oxidation and degradation reactions [29,30,31,32,33,34,35]. Pan-fried samples showed similar but less pronounced effects, likely due to partial lipid migration from the tissue during frying, which reduced the amount of oxidizable substrate retained within the fillet [32,34,35,38]. Boiled samples also retained significant rearing-temperature differences despite moist-heat conditions, indicating that precursor availability in warm-reared fish remained sufficiently high to sustain greater volatile formation even with some leaching into the cooking medium [35,37,38].

#### 3.3.3. Influence of Sex on the Volatile Profile of Cooked Salmon

Sex significantly (*p* = 0.0007) influenced the volatile profile of salmon fillets. In raw samples, sex-related differences were limited. Although the same trend can be seen in all the volatiles, the few significant sex-dependent differences prior to cooking suggest that although females may possess higher precursors, these differences are not strongly reflected in the raw volatile profile.

**Figure 6:**
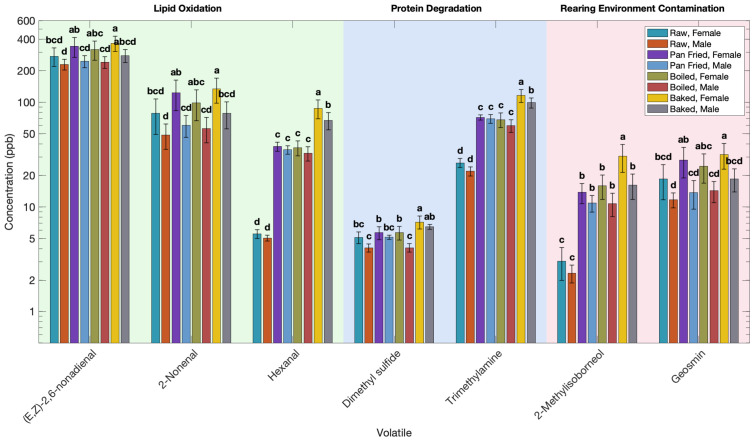
Effect of sex on the volatile profile of cooked salmon. Volatile concentration is plotted on a base-10 logarithmic (log10) scale. Different letters indicate a significant difference in each volatile (*p* < 0.05).

In baked samples, sex effects were most pronounced. Female fish exhibited significantly higher concentrations of lipid-derived volatiles, protein-derived volatiles, and environmental contaminants compared with male fish (Table A5, Figure 6). The strong amplification of sex-related differences during baking is due to sex-dependent differences in lipid content and fatty-acid composition in salmon, with females typically accumulating higher lipid reserves associated with reproductive investment than males as they mature earlier [14,15]. Dry-heat cooking efficiently converts these substrates into volatile compounds through oxidative and degradation pathways [29,30], resulting in greater expression of sex-dependent differences during baking compared with other cooking methods.

In pan-fried samples, female fish exhibited significantly higher concentrations of 25 volatile compounds relative to male fish (Table A5, Figure 6). Compared with baking, the reduced number of sex-dependent differences in fried samples likely reflects partial lipid migration from the fillet during frying, which diminishes the amount of oxidizable substrate retained within the tissue despite high surface temperatures. As a result, sex-related differences in precursor availability are expressed to a lesser extent during frying.

In boiled samples, sex effects were limited, with only six volatile compounds significantly higher in female fish relative to male fish (Table A5, Figure 6). Boiling promoted the leaching of precursors into the cooking medium, thereby reducing the expression of biological variability between sexes.

Sex showed a cooking-dependent influence on the volatile profile of salmon, with differences between female and male fish becoming more pronounced following thermal processing. While sex-related effects were minimal in raw fillets, cooking, particularly under dry-heat conditions, substantially amplified these differences (Table A5, Figure 6), indicating that sex influences the availability and composition of volatile precursors that are selectively expressed during thermal conversion.

#### 3.3.4. Influence of the Presence of Skin (Muscle vs. Skin-On Muscle) on the Volatile Profile of Cooked Salmon

**Figure 7:**
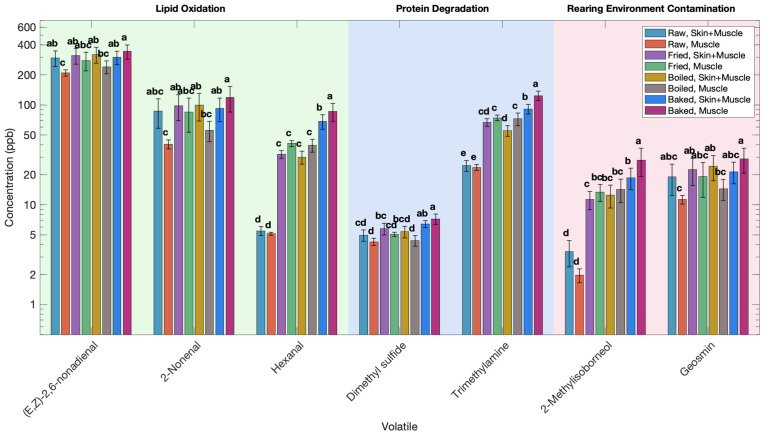
Effect of presence of skin (muscle vs. skin-on muscle) on the volatile profile of cooked salmon. Volatile concentration is plotted on a base-10 logarithmic (log10) scale. Different letters indicate a significant difference in each volatile (*p* < 0.05).

The presence of skin alone did not exert a significant independent effect on the volatile profile of salmon (*p* = 0.1). However, a significant cooking method × skin interaction (*p* = 0.007) demonstrated that the influence of skin depended on cooking conditions (Table A6, Figure 7). This indicates that the effect of skin on volatile formation during cooking is interaction-dependent and controlled primarily by the cooking method. Skin modifies heat transfer, oxygen exposure, and retention of cooking-generated volatile compounds during thermal processing.

In raw samples, skin-on muscle fillets generally exhibited higher volatile concentrations than muscle-only samples (Table A6, Figure 7), consistent with the greater retention of lipid-associated precursors within skin-containing tissues. However, cooking altered this relationship depending on the method applied.

Baking produced the biggest skin-dependent differences. Under baked conditions, muscle-only fillets exhibited higher concentrations of many lipid-derived and protein-derived volatiles compared with skin-on samples (Table A6, Figure 7). This pattern suggests that under prolonged dry-heat exposure, the skin may partially limit oxygen exposure and heat transfer into underlying muscle tissue, thereby reducing thermal oxidation and volatile release relative to directly exposed muscle surfaces.

Pan-fried and boiled samples showed no skin-dependent differences (Table A6, Figure 7), indicating that frying and boiling reduced the influence of skin on volatile formation relative to baking.

## 4. Conclusions

Volatile compound formation in salmon is governed by the combined effects of precursor availability established during growth and the conditions applied during post-harvest processing. The effects of fillet location, storage temperature, and cooking methods on the volatile profile of salmon were evaluated, while considering the influence of pre-harvest and processing factors. Spatial distribution within the fillet introduced significant variability in volatile formation, with the head region consistently exhibiting higher concentrations of volatiles compared to the center and tail. This pattern was associated with higher concentrations of lipid in the head than in the tail.

Storage temperature affected volatile concentrations, likely by determining whether precursors were actively converted into volatile compounds or preserved in their existing biochemical state. Ice storage allowed for the continued lipid oxidation and release of environmental contaminants, resulting in higher volatile concentrations, whereas frozen storage limited these processes and maintained the biochemical state of the fillet. Protein-derived volatiles were less directly influenced by storage alone and were primarily governed by interactions with pre-harvest rearing conditions and the presence of skin. Lower volatile expression was associated with cool-reared fish and muscle-only samples, while warm-reared fish and skin-on samples exhibited greater precursor availability and enhanced the retention and release of volatiles.

Cooking affected the headspace volatile profile, with all cooking methods increasing volatile concentrations of volatiles relative to raw fillets. Dry-heat conditions, particularly baking, produced the highest concentrations due to enhanced lipid oxidation, protein degradation, and retention of environmental contaminants, whereas boiling reduced concentrations through leaching into the cooking water. Lower volatile formation during cooking was associated with cool-reared fish, male fillets, and muscle-only samples, indicating reduced precursor availability and retention compared to warm-reared, female, and skin-on samples.

These findings indicate that volatile concentrations in salmon are influenced by interactions between pre-harvest factors and post-harvest processing. Lipid-rich fillet regions had a stronger contribution to off-odor development, suggesting that targeted antioxidant or preservation strategies may help reduce deterioration and extend salmon shelf life. Integrating optimized rearing conditions with appropriate cooking and storage strategies provides a practical approach to controlling off-odor development, improving flavor quality, and shelf life in salmon.

## Data Availability

The data presented in the study are available in Appendix A. Further inquiries can be directed to the corresponding author.

## Appendix A

**Table A1:** Effect of fillet location on the off-odor volatile compounds (ppb). Different letters indicate a significant difference in each volatile (*p* < 0.05).

Volatile (ppb)	Head	Center	Tail
(E)-2-pentenal	129 ^a^	97.3 ^ab^	80.4 ^b^
(E,Z)-2,6-nonadienal	258 ^a^	176 ^ab^	143 ^b^
1-hexanol	4.23 ^a^	3.43 ^ab^	2.29 ^b^
1-octanol	2.82 ^a^	1.91 ^a^	1.75 ^a^
1-octen-3-ol	687 ^a^	345 ^a^	236 ^a^
1-octen-3-one	1.27 ^a^	1.03 ^ab^	0.81 ^b^
1-pentanol	10.4 ^a^	7.65 ^ab^	6.31 ^b^
1-penten-3-ol	11.7 ^a^	11.2 ^a^	8.56 ^b^
2,3-butanediol	71.5 ^a^	63.5 ^a^	51.2 ^b^
2,4-decadienal	0.88 ^a^	0.71 ^a^	0.63 ^a^
2,4-heptadienal	25.5 ^a^	22.2 ^a^	21.1 ^a^
2-acetyl furan	1.87 ^a^	1.77 ^ab^	1.10 ^b^
2-butenal	1.45 ^a^	1.02 ^a^	1.01 ^a^
2-decanone	1.50 ^a^	0.82 ^a^	0.81 ^a^
2-decenal	2.52 ^a^	1.52 ^a^	1.52 ^a^
2-ethyl-2,5,-dimethylpyrazine	3.92 ^a^	1.79 ^ab^	1.04 ^b^
2-ethylfuran	27.4 ^a^	18.6 ^ab^	14.2 ^b^
2-heptanone	6.31 ^a^	5.38 ^ab^	4.45 ^b^
2-heptenal	13.9 ^a^	8.47 ^ab^	7.09 ^b^
2-hexenal	11.4 ^a^	9.16 ^ab^	6.71 ^b^
2-isopropyl-3-methoxypyrazine	7.94 ^a^	4.92 ^a^	4.11 ^a^
2-methyl naphthalene	8.68 ^a^	5.28 ^ab^	4.24 ^b^
2-methylisoborneol	3.12 ^a^	1.94 ^b^	1.69 ^b^
2-nonanone	1.25 ^a^	0.96 ^ab^	0.56 ^b^
2-nonenal	67.1 ^a^	33.6 ^a^	23.9 ^a^
2-octenal	2.38 ^a^	1.40 ^ab^	1.08 ^b^
2-octene	7.38 ^a^	5.35 ^a^	3.43 ^a^
2-pentanone	1.96 ^a^	1.36 ^b^	1.33 ^b^
2-penten-1-ol	8.60 ^a^	8.25 ^a^	6.31 ^b^
2-pentene	15.4 ^a^	11.3 ^ab^	9.33 ^b^
2-undecanone	0.71 ^a^	0.41 ^a^	0.36 ^a^
3-hexen-1-ol	2676 ^a^	2061 ^ab^	1720 ^b^
3-hexenal	2.50 ^a^	2.22 ^a^	1.88 ^a^
3-methyl-1-butanol	28.5 ^a^	19.2 ^b^	16.6 ^b^
acetic acid	11.5 ^a^	11.2 ^a^	8.70 ^b^
acetoin	4.92 ^a^	4.57 ^a^	3.99 ^a^
acetone	19.0 ^a^	13.9 ^b^	11.7 ^b^
alpha-terpinene	2.25 ^a^	1.61 ^a^	1.54 ^a^
ammonia	466 ^a^	326 ^a^	308 ^a^
beta-caryophyllene	0.85 ^a^	0.51 ^a^	0.32 ^a^
carbon disulfide	1398 ^a^	918 ^a^	714 ^a^
decanal	2.23 ^a^	1.97 ^a^	1.61 ^a^
dimethyl disulfide	7367 ^a^	4873 ^ab^	3894 ^b^
dimethyl sulfide	2.44 ^a^	2.04 ^ab^	1.34 ^b^
dimethylamine	471 ^a^	351 ^ab^	286 ^b^
ethyl acetate	5.47 ^a^	5.08 ^a^	4.43 ^a^
formaldehyde	1279 ^a^	1117 a^b^	1041 ^b^
geosmin	22.3 ^a^	9.29 ^a^	7.16 ^a^
heptanal	4.10 ^a^	2.94 ^b^	2.67 ^b^
heptane	245 ^a^	166 ^ab^	125 ^b^
hexanal	14.6 ^a^	12.1 ^a^	8.97 ^a^
hexanoic acid	1.07 ^a^	1.01 ^a^	0.73 ^a^
hydrogen sulfide	1.24 ^a^	0.55 ^ab^	0.43 ^b^
indole	1.28 ^a^	0.88 ^ab^	0.42 ^b^
isobutyl alcohol	10,883 ^a^	8260 ^ab^	7161 ^b^
methyl mercaptan	12,499 ^a^	9872 ^ab^	8791 ^b^
nonanal	14.5 ^a^	7.67 ^a^	6.14 ^a^
oct-2-en-1-ol	591 ^a^	297 ^a^	204 ^a^
octanal	3.58 ^a^	2.53 ^ab^	1.98 ^b^
octane	55.3 ^a^	34.4 ^ab^	29.4 ^b^
pentanal	13.2 ^a^	10.3 ^b^	9.61 ^b^
pentane	3437 ^a^	2941 ^ab^	2578 ^b^
propanal	17.1 ^a^	14.9 ^a^	14.2 ^a^
trans-2-undecenal	7.74 ^a^	4.02 ^b^	3.60 ^b^
trimethylamine	27.2 ^a^	20.2 ^b^	18.5 ^b^
trimethylpyrazine	22.5 ^a^	12.2 ^a^	9.91 ^a^

**Table A2:** Changes in volatile profile in muscle and skin-on muscle samples reared in cool and warm conditions, harvested at 242 and 301 days (ppb). Different letters indicate a significant difference in each volatile (*p* < 0.05).

Volatiles (ppb)	Day 242, Ice Stored, Cool-Reared Muscle	Day 242, Ice Stored, Cool-Reared Skin + Muscle	Day 242, Frozen, Cool-Reared Muscle	Day 242, Frozen, Cool-Reared Skin + Muscle	Day 301, Ice Stored, Cool-Reared Muscle	Day 301, Ice Stored, Cool-Reared Skin + Muscle	Day 301, Frozen, Cool-Reared Muscle	Day 301, Frozen, Cool-Reared Skin + Muscle	Day 242, Ice Stored, Warm-Reared Muscle	Day 242, Ice Stored, Warm-Reared Skin + Muscle	Day 242, Frozen, Warm-Reared Muscle	Day 242, Frozen, Warm-Reared Skin + Muscle	**Day 301, Ice Stored, Warm-Reared Muscle**	**Day 301, Ice Stored, Warm-Reared Skin + Muscle**	**Day 301, Frozen, Warm-Reared Muscle**	**Day 301, Frozen, Warm-Reared Skin + Muscle**
(E)-2-pentenal	85.2 ^defg^	68.9 ^efg^	90.1 ^cdefg^	100 ^abcdefg^	93.5 ^cdefg^	91.5 ^cdefg^	35.0 ^g^	155 ^abc^	148 ^abcd^	103 ^abcdef^	117 ^abcde^	166 ^a^	150 ^abcd^	163 ^ab^	41.7 ^fg^	97.1 ^bcdefg^
(E,Z)-2,6-nonadienal	141 ^ef^	117 ^ef^	176 ^def^	206 ^cdef^	198 ^def^	196 ^def^	53.2 ^f^	320 ^abcd^	260 ^abcde^	179 ^def^	243 ^bcde^	385 ^ab^	369 ^abc^	415 ^a^	61.6 ^f^	188 ^def^
1-hexanol	3.95 ^bcde^	3.35 ^cdef^	2.65 ^defg^	2.76 ^defg^	2.49 ^efg^	2.84 ^defg^	1.58 ^g^	5.38 ^ab^	6.07 ^a^	4.03 ^bcde^	3.65 ^cde^	4.92 ^abc^	4.20 ^bcd^	4.83 ^abc^	1.81 ^fg^	2.42 ^efg^
1-octanol	4.20 ^abcd^	4.32 ^abc^	1.74 ^ef^	2.57 ^def^	2.75 ^cdef^	3.18 ^bcde^	1.17 ^f^	2.96 ^bcde^	5.11 ^a^	4.24 ^abcd^	2.01 ^ef^	3.14 ^bcde^	4.45 ^ab^	4.89 ^a^	1.59 ^ef^	2.48 ^ef^
1-octen-3-ol	223 ^de^	174 ^e^	302 ^de^	376 c^de^	358 ^cde^	336 ^cde^	62.8 ^e^	990 ^abcd^	552 ^abcde^	310 ^de^	478 ^bcde^	1189 ^ab^	1088 ^abc^	1275 ^a^	71.0 ^e^	369 ^cde^
1-octen-3-one	1.77 ^ab^	1.31 ^abcdef^	0.49 ^gh^	0.57 ^fgh^	1.26 ^abcdefg^	1.55 ^abcd^	0.36 ^h^	1.24 ^abcdefg^	1.96 ^a^	1.39 ^ancde^	0.75 ^efgh^	0.97 ^cdefgh^	1.47 ^abcde^	1.66 ^abc^	0.81 ^defgh^	1.11 ^bcdefgh^
1-pentanol	10.4 ^cd^	11.3 ^bcd^	8.67 ^def^	8.73 ^def^	9.20 ^cde^	9.61 ^cd^	3.79 ^f^	10.9 ^bcd^	15.8 ^ab^	14.0 ^abc^	8.73 ^def^	12.9 ^abcd^	16.5 ^a^	17.1 ^a^	4.22 ^ef^	8.50 ^def^
1-penten-3-ol	21.2 ^ab^	17.6 ^bcd^	12.0 ^def^	8.45 ^fg^	10.2 ^efg^	12.9 ^def^	4.65 ^g^	16.3 ^bcde^	27.9 ^a^	20.1 ^bc^	10.1 ^efg^	13.8 ^cdef^	18.4 ^bcd^	21.1 ^ab^	4.13 ^g^	6.95 ^fg^
2,3-butanediol	64.5 ^cdef^	78.0 ^abcd^	73.7 ^bcde^	48.7 ^efgh^	42.6 ^fgh^	63.7 ^cdefg^	28.4 ^h^	87.3 ^abc^	98.8 ^ab^	89.6 ^abc^	64.5 ^cdef^	67.8 ^cdef^	66.6 ^cdef^	103 ^a^	35.0 ^gh^	50.7 ^defgh^
2,4-decadienal	1.06 ^abc^	0.90 ^abcd^	0.38 ^d^	0.59 ^cd^	0.79 ^bcd^	0.83 ^abcd^	0.84 ^abcd^	0.93 ^abcd^	1.45 ^a^	1.18 ^abc^	0.62 ^cd^	0.78 ^bcd^	0.64 ^cd^	1.31 ^ab^	0.83 ^abcd^	0.66 ^cd^
2,4-heptadienal	41.2 ^b^	29.9 ^b^	22.8 ^b^	17.6 ^b^	17.2 ^b^	16.6 ^b^	12.7 ^b^	37.0 ^b^	95.5 ^a^	37.4 ^b^	20.2 ^b^	23.1 ^b^	18.7 ^b^	21.4 ^b^	14.8 ^b^	12.3 ^b^
2-acetyl furan	1.57 ^bcde^	1.83 ^bcd^	1.19 ^de^	1.29 ^cde^	1.30 ^cde^	1.21 ^de^	0.95 ^e^	1.99 ^bcd^	3.22 ^a^	2.40 ^ab^	1.61 ^bcde^	1.87 ^bcd^	2.20 ^b^	2.11 ^bc^	0.84 ^e^	1.92 ^bcd^
2-butenal	2.48 ^bc^	2.68 ^b^	1.72 ^cde^	1.84 ^cd^	1.32 ^def^	1.16 ^def^	0.65 ^f^	1.71 ^cde^	4.00 ^a^	2.66 ^b^	1.80 ^cd^	2.24 ^bc^	1.37 ^def^	2.40 ^bc^	0.74 ^f^	0.93 ^ef^
2-decanone	2.31 ^ab^	2.28 ^abc^	1.04 ^cde^	0.92 ^de^	1.50 ^abcde^	1.53 ^abcde^	0.51 ^e^	1.81 ^abcd^	2.52 ^a^	1.84 ^abcd^	0.83 ^de^	1.11 ^bcde^	1.17 ^bcde^	2.24 ^abc^	0.96 ^de^	1.10 ^bcde^
2-decenal	5.68 ^b^	2.93 ^cde^	1.97 ^def^	1.97 ^def^	2.01 ^def^	2.31 ^def^	0.96 ^f^	2.49 ^def^	7.54 ^a^	3.25 ^cd^	2.45 ^def^	2.93 ^cde^	2.99 ^cde^	4.44 ^bc^	1.57 ^ef^	2.22 ^def^
2-ethyl-2,5,-dimethylpyrazine	2.65 ^bcd^	1.89 ^cd^	2.02 ^bcd^	1.71 ^cd^	2.00 ^bcd^	2.60 ^cd^	0.54 ^d^	5.14 ^ab^	4.36 ^abc^	2.30 ^bcd^	2.20 ^bcd^	4.31 ^abc^	4.54 ^abc^	7.00 ^a^	0.59 ^d^	2.62 ^bcd^
2-ethylfuran	15.0 ^de^	10.4 ^de^	15.8 ^de^	18.5 ^cde^	19.7 ^bcde^	17.4 ^cde^	5.86 ^e^	36.4 ^abc^	26.2 ^abcd^	16.2 ^cde^	22.5 ^abcde^	38.9 ^ab^	40.3 ^a^	41.0 ^a^	6.02 ^de^	17.1 ^cde^
2-heptanone	4.93 ^bcd^	3.26 ^d^	4.16 ^cd^	5.09 ^bcd^	4.74 ^bcd^	5.40 ^abc^	4.40 ^cd^	6.74 ^ab^	4.78 ^bcd^	4.51 ^cd^	5.10 ^bcd^	6.82 ^ab^	7.38 ^a^	6.65 ^ab^	4.84 ^bcd^	5.26 ^abcd^
2-heptenal	10.9 ^bc^	9.22 ^bc^	8.05 ^bc^	9.73 ^bc^	8.34 ^bc^	9.18 ^bc^	3.93 ^c^	17.0 ^ab^	17.3 ^ab^	10.6 ^bc^	9.25 ^bc^	18.2 ^ab^	17.5 ^ab^	23.7 ^a^	4.32 ^c^	10.6 ^bc^
2-hexenal	11.7 ^bcd^	10.1 ^cd^	7.30 c^d^	7.60 ^cd^	8.97 ^cd^	8.53 ^cd^	4.65 ^d^	11.8 ^abcd^	18.4 ^ab^	12.2 ^abc^	10.2 ^cd^	17.7 ^ab^	19.1 ^a^	18.6 ^ab^	6.95 ^cd^	10.1 ^cd^
2-isopropyl-3-methoxypyrazine	21.0 ^b^	4.82 ^cde^	4.38 ^de^	5.44 ^cde^	6.01 ^cde^	5.48 ^cde^	1.63 ^e^	9.57 ^cd^	29.2 ^a^	7.04 ^cde^	7.46 ^cde^	10.9 ^c^	10.2 ^cd^	10.6 ^cd^	2.10 ^e^	6.25 ^cde^
2-methyl naphthalene	7.90 ^bcd^	10.5 ^bcd^	4.29 ^cd^	9.23 ^bcd^	12.5 ^bcd^	14.7 ^bc^	2.57 ^d^	10.6 ^bcd^	17.5 ^b^	14.1 ^bc^	6.67 ^cd^	14.1 ^bc^	31.7 ^a^	36.1 ^a^	3.38 ^d^	6.58 ^cd^
2-methylisoborneol	3.22 ^bcde^	2.63 ^bcde^	2.12 ^cde^	1.98 ^cde^	2.37 ^cde^	3.03 ^bcde^	0.86 ^e^	3.37 ^bcd^	5.00 ^ab^	2.91 ^bcde^	1.81 ^de^	4.83 ^ab^	4.36 ^abc^	6.16 ^a^	1.70 ^de^	2.70 ^bcde^
2-nonanone	2.28 ^ab^	2.34 ^a^	0.76 ^d^	0.99 ^d^	1.36 ^bcd^	2.13 ^abc^	0.69 ^d^	0.77 ^d^	2.47 ^a^	2.57 ^a^	0.89 ^d^	1.16 ^d^	1.20 ^cd^	2.38 ^a^	0.61 ^d^	1.13 ^d^
2-nonenal	25.7 ^bc^	17.9 ^bc^	31.2 ^bc^	41.1 ^bc^	41.6 ^bc^	40.3 ^bc^	7.81 ^c^	95.0 ^ab^	50.8 ^bc^	31.1 ^bc^	49.7 ^bc^	132 ^a^	131 ^a^	146 ^a^	8.19 ^c^	37.3 ^bc^
2-octenal	1.61 ^cde^	1.47 ^cde^	1.00 ^de^	0.99 ^de^	0.83 ^e^	1.34 ^cde^	0.93 ^e^	3.44 ^a^	2.81 ^ab^	2.14 ^bcd^	1.15 ^cde^	1.63 ^cde^	1.74 ^bcde^	2.22 ^bc^	1.20 ^cde^	1.29 ^cde^
2-octene	5.58 ^cd^	5.52 ^cd^	5.58 ^cd^	5.61 ^cd^	5.37 ^cd^	5.94 ^cd^	2.28 ^d^	9.88 ^abc^	9.83 ^abc^	6.71 ^bcd^	7.19 ^bcd^	12.0 ^ab^	12.6 ^ab^	15.2 ^a^	2.44 ^d^	4.85 ^cd^
2-pentanone	1.48 ^bcdef^	1.57 ^abcde^	1.25 ^cdefgh^	0.81 ^fgh^	1.24 ^defgh^	1.09 ^efgh^	0.64 ^h^	2.27 ^a^	1.96 ^abcd^	2.11 ^ab^	1.28 ^cdefgh^	1.25 ^defgh^	1.98 ^abc^	1.95 ^abcd^	0.73 ^gh^	1.47 ^bcdefg^
2-penten-1-ol	15.7 ^ab^	12.9 ^bcd^	8.82 ^def^	6.23 ^fg^	7.49 ^efg^	9.47 ^def^	3.43 ^g^	12.0 ^bcde^	20.6 ^a^	14.8 ^bc^	7.44 ^efg^	10.2 ^cdef^	13.6 ^bcd^	15.6 ^ab^	3.05 ^g^	5.13 ^fg^
2-pentene	15.4 ^cd^	16.7 ^bcd^	12.8 ^def^	12.9 ^def^	13.6 ^cde^	14.2 ^cd^	5.60 ^f^	16.2 ^bcd^	23.4 ^ab^	20.7 ^abc^	12.9 ^def^	19.1 ^abcd^	24.4 ^a^	25.4 ^a^	6.23 ^ef^	12.6 ^def^
2-undecanone	2.06 ^ab^	1.21 ^cdef^	0.24 ^g^	0.70 ^defg^	1.46 ^bcde^	1.49 ^bcd^	0.66 ^efg^	0.51 ^fg^	1.38 ^bcde^	2.66 ^a^	1.08 ^cdef^	0.89 ^cdefg^	1.39 ^bcde^	1.60 ^bc^	0.48 ^fg^	0.97 ^cdefg^
3-hexen-1-ol	1882 ^bcde^	1635 ^cde^	1826 ^bcde^	2037 ^abcde^	1883 ^bcde^	1823 ^cde^	897 ^e^	3184 ^a^	3080 ^ab^	2241 ^abcd^	2342 ^abc^	3231 ^a^	3022 ^ab^	3277 ^a^	992 ^de^	2043 ^abcde^
3-hexenal	5.86 ^ab^	5.30 ^abc^	3.43 ^cde^	3.23 ^de^	4.93 ^abcd^	4.46 ^bcd^	1.75 ^e^	2.30 ^e^	6.52 ^a^	5.63 ^ab^	3.17 ^de^	5.39 ^abc^	5.98 ^ab^	6.11 ^ab^	2.04 ^e^	2.22 ^e^
3-methyl-1-butanol	20.5 ^bc^	17.7 ^c^	21.1 ^bc^	16.0 ^c^	19.4 ^bc^	17.1 ^c^	12.5 ^c^	35.4 ^ab^	44.2 ^a^	21.2 ^bc^	19.2 ^bc^	26.7 ^bc^	26.4 ^bc^	34.8 ^ab^	22.0 ^bc^	19.5 ^bc^
acetic acid	12.7 ^bc^	17.0 ^ab^	11.4 ^bcd^	7.93 ^cdef^	6.24 ^def^	10.8 ^cd^	4.08 ^f^	11.5 ^bcd^	20.6 ^a^	20.4 ^a^	10.6 ^cde^	11.9 ^bcd^	9.32 ^cdef^	17.0 ^ab^	4.76 ^ef^	10.4 ^cde^
acetoin	4.38 ^bcde^	4.11 ^cde^	4.18 ^cde^	3.78 ^de^	4.16 ^cde^	4.18 ^cde^	3.17 ^e^	5.38 ^abcd^	5.96 ^ab^	5.49 ^abc^	4.28 ^cde^	4.81 ^bcde^	4.93 ^bcd^	6.64 ^a^	3.26 ^e^	4.12 ^cde^
acetone	16.4 ^bcde^	16.7 ^bcde^	12.8 ^cde^	12.3 ^def^	10.9 ^efg^	12.7 ^cde^	5.46 ^g^	18.4 ^abc^	23.7 ^a^	17.5 ^bcd^	14.8 ^bcde^	18.8 ^ab^	16.3 ^bcde^	19.6 ^ab^	6.52 ^fg^	18.0 ^abcd^
alpha-terpinene	2.04 ^abcde^	2.13 ^abcde^	1.90 ^abcde^	1.41 ^cde^	1.41 ^cde^	1.94 ^abcde^	0.91 ^e^	2.67 ^ab^	2.99 ^a^	2.58 ^abc^	1.62 ^bcde^	1.94 ^abcde^	2.17 ^abcd^	2.72 ^ab^	1.25 ^de^	1.41 ^cde^
ammonia	461 ^c^	476 ^c^	502 ^c^	619 ^bc^	619 ^bc^	519 ^c^	375 ^c^	587 ^c^	595 ^bc^	500 ^c^	552 ^c^	1094 ^a^	957 ^a^	922 ^ab^	381 ^c^	327 ^c^
beta-caryophyllene	2.04 ^abcd^	3.31 ^a^	0.86 ^def^	0.87 ^def^	0.95 ^def^	1.60 ^bcde^	0.29 ^f^	1.17 ^def^	2.48 ^abc^	2.73 ^ab^	0.54 ^ef^	0.96 ^def^	1.34 ^cdef^	1.94 ^bcd^	0.21 ^f^	0.51 ^ef^
carbon disulfide	771 ^cd^	588 ^d^	847 ^cd^	1016 ^cd^	1180 ^bcd^	1123 ^cd^	329 ^d^	1896 ^abc^	1335 ^bcd^	880 ^cd^	1185 ^bcd^	2268 ^ab^	2579 ^a^	2715 ^a^	348 ^d^	848 ^cd^
decanal	2.84 ^abcd^	2.61 ^abcde^	1.37 ^efgh^	1.89 ^bcdefgh^	1.88 ^cdefgh^	1.78 ^defgh^	1.17 ^gh^	2.34 ^abcdefg^	3.14 ^ab^	3.52 ^a^	1.27 f^gh^	2.22 ^bcdefgh^	2.43 ^abcdef^	3.13 ^abc^	1.00 ^h^	2.06 ^bcdefgh^
dimethyl disulfide	3736 ^d^	2783 ^d^	4240 ^d^	5036 ^cd^	5311 ^bcd^	4854 ^cd^	1500 ^d^	9755 ^abc^	6625 ^abcd^	4308 ^d^	5724 ^bcd^	10,340 a^b^	11,023 ^a^	11,159 ^a^	1718 ^d^	4693 ^cd^
dimethyl sulfide	3.28 ^def^	3.53 ^def^	3.75 ^def^	3.77 ^def^	4.56 ^bcd^	4.87 ^bcd^	1.33 ^g^	2.40 ^fg^	4.69 ^bcd^	4.14 ^cde^	4.78 ^bcd^	6.12 ^ab^	5.81 ^abc^	7.31 ^a^	1.02 ^g^	2.62 ^efg^
dimethylamine	287 ^bcdef^	278 ^bcdef^	244 ^cdef^	256 ^cdef^	218 ^def^	298 ^bcdef^	77.2 ^f^	416 ^abcd^	569 ^ab^	450 ^abcd^	373 ^abcde^	632 ^a^	495 ^abcd^	648 ^a^	84.1 ^ef^	520 ^abc^
ethyl acetate	4.87 ^bcde^	4.56 ^cde^	4.64 ^cde^	4.20 ^de^	4.62 ^cde^	4.64 ^cde^	3.52 ^e^	5.98 ^abcd^	6.63 ^ab^	6.10 ^abc^	4.75 ^cde^	5.35 ^bcde^	5.48 ^cd^	7.38 ^a^	3.62 ^e^	4.58 ^cde^
formaldehyde	1339 ^cde^	1197 ^de^	1107 ^def^	1190 ^de^	1274 ^cde^	1245 ^cde^	766 ^f^	1444 ^abcd^	1603 ^abc^	1389 ^bcde^	1236 ^cde^	1610 ^abc^	1736 ^ab^	1775 ^a^	806 ^f^	1062 ^ef^
geosmin	9.54 ^bc^	7.54 ^bc^	9.14 ^bc^	9.14 ^bc^	9.43 ^bc^	9.57 ^bc^	3.04 ^c^	31.2 ^ab^	17.0 ^abc^	8.83 ^bc^	13.5 ^abc^	28.8 ^ab^	30.0 ^ab^	35.8 ^a^	2.69 ^c^	12.9 ^abc^
heptanal	4.16 ^cde^	4.60 ^cde^	3.08 ^def^	4.41 ^cde^	4.80 ^cde^	5.18 ^c^	1.87 ^f^	4.75 ^cde^	5.87 ^bc^	5.27 ^c^	3.02 ^def^	4.97 ^cd^	7.39 ^b^	9.72 ^a^	2.11 ^f^	2.94 ^ef^
heptane	185 ^cdef^	184 ^cdef^	240 ^bcde^	247 ^bcd^	227 ^bcdef^	170 ^cdef^	54.4 ^f^	327 ^bcd^	318 ^bcd^	197 ^bcdef^	258 ^bcd^	523 ^a^	338 ^bc^	366 ^ab^	66.9 ^ef^	153 ^def^
hexanal	8.20 ^b^	8.41 ^b^	4.81 ^b^	4.75 ^b^	6.15 ^b^	4.78 ^b^	3.16 ^b^	18.1 ^a^	21.0 ^a^	9.48 ^b^	5.45 ^b^	6.20 ^b^	7.04 ^b^	8.22 ^b^	6.34 ^b^	7.94 ^b^
hexanoic acid	1.15 ^bcd^	1.66 ^abc^	0.89 ^cd^	0.90 ^bcd^	1.20 ^bcd^	1.35 ^abcd^	0.57 ^d^	1.21 ^bcd^	1.62 ^abc^	1.68 ^abc^	0.82 ^cd^	1.52 ^abc^	1.82 ^ab^	2.26 ^a^	0.47 ^d^	1.03 ^bcd^
hydrogen sulfide	2.18 ^abc^	1.83 ^bcdef^	0.50 ^g^	0.93 ^defg^	0.80 ^efg^	2.08 a^bcd^	0.26 ^g^	1.32 ^cdefg^	2.88 ^ab^	1.85 ^bcdef^	0.69 ^fg^	0.83 ^efg^	1.87 ^bcde^	3.07 ^a^	0.43 ^g^	1.16 ^cdefg^
indole	1.70 ^abc^	1.77 ^ab^	0.68 ^cd^	0.94 ^bcd^	1.04 ^bcd^	0.80 ^bcd^	0.48 ^d^	1.35 ^bcd^	1.64 ^abc^	1.62 ^abc^	1.21 ^bcd^	0.95 ^bcd^	1.79 ^ab^	2.40 ^a^	0.45 ^d^	1.22 ^bcd^
isobutyl alcohol	7998 ^cde^	6513 ^de^	7598 ^de^	8588 ^cde^	9266 ^bcde^	8816 ^bcde^	4166 ^e^	13,430 ^abc^	11,531 ^abcd^	8740 ^cde^	9336 ^bcde^	14,410 ^ab^	15,832 ^a^	15,955 ^a^	4606 ^e^	7894 ^cde^
methyl mercaptan	9424 ^cdef^	7805 ^def^	8406 ^def^	9425 ^cdef^	10,396 ^bcde^	9820 ^bcdef^	4772 ^f^	15,111 ^ab^	12,978 ^abcd^	10,135 ^bcdef^	10,122 ^bcdef^	14,923 ^abc^	16,855 ^a^	16,744 ^a^	5198 ^ef^	9376 ^def^
nonanal	8.72 ^de^	11.4 ^cde^	8.64 ^de^	13.4 ^cde^	16.3 ^cde^	17.8 ^cde^	2.54 ^e^	20.1 ^cd^	16.0 ^cde^	13.3 ^cde^	11.8 ^cde^	27.3 ^bc^	41.7 ^ab^	44.3 ^a^	2.54 ^e^	8.35 ^de^
oct-2-en-1-ol	193 ^de^	151 ^e^	261 ^de^	324 ^cde^	309 ^cde^	290 ^cde^	54.4 ^e^	852 ^abcd^	476 ^abcde^	268 ^de^	412 ^bcde^	1024 ^ab^	938 ^abc^	1100 ^a^	61.5 ^e^	318 ^cde^
octanal	3.67 ^cdef^	3.49 ^cdefg^	2.07 ^fgh^	2.60 ^efgh^	3.62 ^cdef^	3.33 ^cdefg^	1.25 ^h^	4.31 ^bcd^	6.48 ^a^	4.06 ^bcde^	2.36 ^fgh^	2.66 ^defgh^	4.69 ^bc^	5.44 ^ab^	1.89 ^gh^	2.55 ^efgh^
octane	42.5 ^bcd^	34.3 ^bcd^	31.8 ^cd^	40.3 ^bcd^	34.4 ^bcd^	36.4 ^bcd^	16.9 ^d^	66.8 ^abc^	58.1 ^abc^	40.5 ^bcd^	35.4 ^bcd^	70.0 ^ab^	64.6 ^abc^	85.2 ^a^	19.2 ^d^	43.4 ^bcd^
pentanal	8.50 ^bc^	8.07 ^bcd^	9.08 ^b^	4.22 ^e^	4.01 ^e^	6.02 ^bcde^	4.39 ^e^	18.0 ^a^	8.97 ^b^	7.90 ^bcd^	6.79 ^bcde^	5.43 ^cde^	5.19 ^cde^	7.90 ^bcd^	4.99 ^de^	8.51 ^bc^
pentane	3353 ^bc^	4042 ^b^	3518 ^bc^	2644 ^cd^	2601 ^cd^	3594 ^bc^	1526 ^d^	4297 ^b^	4612 ^b^	4149 ^b^	3239 ^bc^	4166 ^b^	4344 ^b^	6058 ^a^	1554 ^d^	2396 ^cd^
propanal	27.7 ^b^	20.1 ^b^	15.3 ^b^	11.8 ^b^	11.5 ^b^	11.2 ^b^	8.52 ^b^	24.9 ^b^	64.2 ^a^	25.2 ^b^	13.6 ^b^	15.6 ^b^	12.6 ^b^	14.4 ^b^	9.97 ^b^	8.30 ^b^
trans-2-undecenal	7.28 ^bcde^	7.78 ^bcde^	5.99 ^cde^	5.44 ^de^	8.11 ^bcde^	8.21 ^bcde^	2.14 ^e^	9.52 ^bcde^	11.4 ^abcd^	8.53 ^bcde^	7.27 ^bcde^	13.5 ^abc^	14.7 ^ab^	17.8 ^a^	2.60 ^e^	5.81 ^cde^
trimethylamine	27.5 ^bc^	25.5 ^bc^	22.6 ^bc^	20.8 ^c^	23.6 ^bc^	22.5 ^bc^	22.6 ^bc^	30.6 ^abc^	44.2 ^a^	29.6 ^bc^	24.9 ^bc^	28.6 ^bc^	30.4 ^abc^	32.2 ^abc^	35.3 ^ab^	20.4 ^c^
trimethylpyrazine	9.66 ^e^	7.36 ^e^	10.8 ^de^	14.3 ^de^	16.6 ^de^	16.2 ^de^	3.44 ^e^	31.1 ^abcd^	19.7 ^bcde^	11.6 ^de^	18.3 ^cde^	37.5 ^abc^	40.2 ^ab^	44.7 ^a^	4.29 ^e^	13.0 ^de^

**Table A3:** Effect of cooking methods on the volatile profile of salmon fillets (ppb). Different letters indicate a significant difference in each volatile (*p* < 0.05).

Volatile (ppb)	Baked	Boiled	Fried	Raw
(E,Z)-2,6-nonadienal	322 ^a^	280 ^ab^	294 ^ab^	252 ^b^
€-2-pentenal	145 ^a^	129 ^ab^	134 ^ab^	118 ^b^
1-hexanol	10.9 ^a^	6.94 ^b^	6.32 ^b^	3.49 ^c^
1-octanol	5.49 ^a^	3.52 ^b^	4.00 ^b^	2.37 ^c^
1-octen-3-ol	925 ^a^	727 ^ab^	818 ^ab^	586 ^b^
1-octen-3-one	1.45 ^a^	1.02 ^b^	1.01 ^b^	0.70 ^b^
1-pentanol	23.3 ^a^	14.3 ^b^	15.7 ^b^	9.76 ^c^
1-penten-3-ol	77.4 ^a^	40.8 ^b^	43.4 ^b^	11.1 ^c^
2-acetyl furan	2.61 ^a^	2.17 ^ab^	2.09 ^b^	1.49 ^c^
2-butenal	7.98 ^a^	5.36 ^b^	5.16 ^b^	1.90 ^c^
2-decanone	1.18 ^ab^	1.50 ^a^	0.89 ^b^	0.97 ^b^
2-decenal	4.31 ^a^	3.15 ^bc^	3.62 ^ab^	2.33 ^c^
2-ethyl-2,5,-dimethylpyrazine	4.40 ^a^	2.81 ^b^	3.80 ^ab^	2.56 ^b^
2-ethylfuran	34.7 ^a^	27.5 ^bc^	30.9 ^ab^	23.9 ^c^
2-heptanone	6.13 ^ab^	6.23 ^a^	5.38 ^ab^	5.29 ^b^
2-heptenal	32.6 ^a^	17.4 ^bc^	20.5 ^b^	11.3 ^c^
2-hexenal	21.5 ^a^	13.6 ^bc^	15.3 ^b^	10.7 ^c^
2-isopropyl-3-methoxypyrazine	16.7 ^a^	9.72 ^bc^	11.68 ^b^	7.05 ^c^
2-methyl naphthalene	17.8 ^a^	11.3 ^bc^	14.7 ^ab^	8.58 ^c^
2-methylisoborneol	23.4 ^a^	13.4 ^b^	12.4 ^b^	2.69 ^c^
2-nonanone	1.38 ^a^	1.11 ^ab^	1.06 ^ab^	0.95 ^b^
2-nonenal	106 ^a^	77.6 ^ab^	91.6 ^ab^	63.6 ^b^
2-octenal	2.43 ^a^	1.78 ^b^	1.97 ^b^	1.19 ^c^
2-octene	11.5 ^a^	10.2 ^ab^	9.94 ^ab^	7.60 ^b^
2-pentanone	2.49 ^a^	1.69 ^b^	1.83 ^b^	1.15 ^c^
2-penten-1-ol	57.0 ^a^	30.1 ^b^	32.0 ^b^	8.17 ^c^
2-pentene	34.5 ^a^	21.2 ^b^	23.2 ^b^	14.4 ^c^
2-undecanone	0.83 ^a^	0.80 ^a^	0.78 ^a^	0.73 ^a^
2,3-butanediol	86.0 ^a^	34.9 ^c^	70.9 ^b^	63.7 ^b^
2,4-decadienal	2.72 ^a^	1.61 ^b^	1.70 ^b^	0.59 ^c^
2,4-heptadienal	359 ^a^	204 ^b^	198 ^b^	20.9 ^c^
3-hexen-1-ol	2889 ^a^	2561 ^ab^	2673 ^ab^	2359 ^b^
3-hexenal	8.68 ^a^	5.02 ^bc^	6.01 ^b^	3.81 ^c^
3-methyl-1-butanol	196 ^a^	102 ^b^	107 ^b^	20.7 ^c^
acetic acid	13.4 ^a^	6.98 ^c^	10.9 ^b^	10.4 ^b^
acetoin	5.05 ^a^	4.06 ^b^	5.06 ^a^	4.26 ^b^
acetone	40.9 ^a^	22.8 ^c^	28.0 ^b^	14.7 ^d^
alpha-terpinene	2.41 ^a^	2.13 ^ab^	2.08 ^ab^	1.72 ^b^
ammonia	978 ^a^	773 ^b^	977 ^a^	691 ^b^
beta-caryophyllene	0.49 ^a^	0.65 ^a^	0.88 ^a^	0.81 ^a^
carbon disulfide	2066 ^a^	1596 ^bc^	1851 ^ab^	1329 ^c^
decanal	2.12 ^ab^	1.81 ^ab^	2.21 ^a^	1.69 ^b^
dimethyl disulfide	8790 ^a^	7352 ^b^	8001 ^ab^	6335 ^b^
dimethyl sulfide	6.83 ^a^	4.88 ^b^	5.41 ^b^	4.60 ^b^
dimethylamine	459 ^a^	396 ^a^	428 ^a^	376 ^a^
ethyl acetate	5.61 ^a^	4.51 ^b^	5.62 ^a^	4.73 ^b^
formaldehyde	1502 ^a^	1385 ^ab^	1436 ^a^	1286 ^b^
geosmin	25.1 ^a^	19.4 ^ab^	20.8 ^ab^	15.1 ^b^
heptanal	7.87 ^a^	5.39 ^c^	6.43 ^b^	3.87 ^d^
heptane	1541 ^a^	496 ^c^	757 ^b^	317 ^c^
hexanal	77.2 ^a^	34.8 ^b^	36.6 ^b^	5.30 ^c^
hexanoic acid	2.04 ^a^	1.71 ^ab^	1.53 ^b^	1.04 ^c^
hydrogen sulfide	2.93 ^a^	1.31 ^a^	0.86 ^a^	0.74 ^a^
indole	1.41 ^a^	1.11 ^a^	1.46 ^a^	0.95 ^a^
isobutyl alcohol	12,764 ^a^	11,149 ^ab^	11,906 ^a^	9983 ^b^
methyl mercaptan	13,564 ^a^	11,885 ^ab^	12,656 ^a^	10,719 ^b^
nonanal	26.1 ^a^	19.0 ^bc^	22.6 ^ab^	15.3 ^c^
oct-2-en-1-ol	797 ^a^	627 ^ab^	705 ^ab^	505 ^b^
octanal	6.50 ^a^	3.59 ^b^	4.20 ^b^	2.42 ^c^
octane	64.0 ^a^	49.8 ^ab^	57.6 ^ab^	44.4 ^b^
pentanal	50.7 ^a^	13.7 ^c^	23.6 ^b^	6.38 ^d^
pentane	4834 ^a^	2151 ^d^	3987 ^b^	3392 ^c^
propanal	241 ^a^	137 ^b^	133 ^b^	14.1 ^c^
trans-2-undecenal	13.5 ^a^	11.3 ^ab^	13.0 ^a^	8.05 ^b^
trimethylamine	108 ^a^	64.0 ^b^	70.5 ^b^	24.2 ^c^
trimethylpyrazine	31.6 ^a^	25.0 ^ab^	27.7 ^ab^	20.2 ^b^

**Table A4:** Effect of pre-harvest rearing conditions on the volatile profile of cooked salmon (ppb). Different letters indicate a significant difference in each volatile (*p* < 0.05).

Volatile (ppb)	Raw, Cool	Raw, Warm	Fried, Cool	Fried, Warm	Boiled, Cool	Boiled, Warm	Baked, Cool	Baked, Warm
(E)-2-pentenal	92.7 ^c^	144 ^b^	91.4 ^c^	177 ^a^	94.7 ^c^	164 ^ab^	103 ^c^	188 ^a^
(E,Z)-2,6-nonadienal	184 ^c^	321 ^b^	178 ^c^	411 ^a^	183 ^c^	377 ^ab^	214 ^c^	430 ^a^
1-hexanol	2.66 ^e^	4.33 ^d^	5.31 ^d^	7.34 ^c^	4.62 ^d^	9.27 ^b^	7.17 ^c^	14.6 ^a^
1-octanol	2.03 ^d^	2.71 ^cd^	2.86 ^cd^	5.14 ^b^	2.14 ^d^	4.90 ^b^	3.46 ^c^	7.51 ^a^
1-octen-3-ol	296 ^c^	877 ^b^	271 ^c^	1364 ^a^	305 ^c^	1148 ^ab^	376 ^c^	1475 ^a^
1-octen-3-one	0.50 ^d^	0.90 ^bcd^	0.71 ^cd^	1.32 ^b^	0.69 ^cd^	1.36 ^b^	1.02 ^bc^	1.88 ^a^
1-pentanol	8.49 ^e^	11.0 ^de^	11.0 ^de^	20.4 ^b^	10.7 ^de^	18.0 ^bc^	14.3 ^cd^	32.3 ^a^
1-penten-3-ol	10.1 ^e^	12.1 ^e^	30.9 ^cd^	56.0 ^b^	23.6 ^de^	58.0 ^b^	46.0 ^bc^	109 ^a^
2,3-butanediol	59.7 ^cd^	67.7 ^c^	59.2 ^cd^	82.6 ^b^	20.0 ^e^	49.7 ^d^	55.6 ^cd^	116 ^a^
2,4-decadienal	0.47 ^d^	0.71 ^d^	1.08 ^cd^	2.31 ^b^	0.80 ^d^	2.43 ^b^	1.56 ^c^	3.88 ^a^
2,4-heptadienal	20.2 ^f^	21.6 ^f^	185 ^de^	212 ^cd^	131 ^e^	276 ^b^	259 ^bc^	459 ^a^
2-acetyl furan	1.25 ^d^	1.73 ^cd^	1.61 ^cd^	2.56 ^ab^	1.68 ^cd^	2.66 ^ab^	2.05 ^bc^	3.18 ^a^
2-butenal	1.75 ^g^	2.04 ^fg^	4.14 ^de^	6.19 ^bc^	3.54 ^ef^	7.18 ^b^	5.33 ^cd^	10.6 ^a^
2-decanone	1.02 ^b^	0.93 ^b^	0.70 ^b^	1.07 ^b^	0.88 ^b^	2.12 ^a^	1.08 ^b^	1.27 ^b^
2-decenal	1.94 ^c^	2.73 ^c^	2.35 ^c^	4.90 ^ab^	2.13 ^c^	4.18 ^b^	2.61 ^c^	6.01 ^a^
2-ethyl-2,5,-dimethylpyrazine	1.59 ^c^	3.53 ^bc^	1.73 ^c^	5.87 ^a^	1.73 ^c^	3.90 ^b^	2.52 ^bc^	6.29 ^a^
2-ethylfuran	16.3 ^e^	31.5 ^cd^	17.4 ^e^	44.4 ^ab^	17.9 ^e^	37.1 ^bc^	21.9 ^de^	47.4 ^a^
2-heptanone	4.49 ^c^	6.09 ^ab^	4.59 ^c^	6.17 ^ab^	5.41 ^bc^	7.05 ^a^	5.61 ^bc^	6.64 ^ab^
2-heptenal	8.37 ^c^	14.2 ^c^	10.5 ^c^	30.5 ^b^	9.12 ^c^	25.7 ^b^	13.4 ^c^	51.8 ^a^
2-hexenal	6.94 ^e^	14.5 ^cd^	9.32 ^de^	21.2 ^b^	8.00 ^e^	19.1 ^bc^	10.9 ^de^	32.1 ^a^
2-isopropyl-3-methoxypyrazine	4.40 ^d^	9.70 ^cd^	5.17 ^d^	18.2 ^b^	5.23 ^d^	14.2 ^bc^	7.54 ^d^	25.8 ^a^
2-methyl naphthalene	6.51 ^c^	10.6 ^c^	8.80 ^c^	20.5 ^b^	5.72 ^c^	16.8 ^b^	9.37 ^c^	26.2 ^a^
2-methylisoborneol	1.93 ^d^	3.44 ^cd^	7.23 ^cd^	17.5 ^b^	6.17 ^cd^	20.6 ^b^	9.52 ^c^	37.2 ^a^
2-nonanone	0.76 ^c^	1.14 ^abc^	0.95 ^bc^	1.17 ^abc^	0.81 ^bc^	1.40 ^ab^	1.10 ^abc^	1.65 ^a^
2-nonenal	31.2 ^c^	96.0 ^b^	31.7 ^c^	152 ^a^	33.0 ^c^	122 ^ab^	44.1 ^c^	168 ^a^
2-octenal	0.99 ^c^	1.40 ^c^	1.55 ^c^	2.40 ^b^	1.08 ^c^	2.48 ^b^	1.56 ^c^	3.30 ^a^
2-octene	5.31 ^cd^	9.89 ^bc^	4.38 ^d^	15.5 ^a^	6.35 ^cd^	14.0 ^ab^	6.71 ^cd^	16.3 ^a^
2-pentanone	1.02 ^d^	1.27 ^d^	1.41 ^cd^	2.25 ^b^	1.24 ^d^	2.13 ^b^	1.88 ^bc^	3.10 ^a^
2-penten-1-ol	7.42 ^e^	8.92 ^e^	22.7 ^cd^	41.3 ^b^	17.4 ^de^	42.8 ^b^	33.9 ^bc^	80.2 ^a^
2-pentene	12.6 ^e^	16.3 ^de^	16.2 ^de^	30.2 ^b^	15.8 ^de^	26.6 ^bc^	21.1 ^cd^	47.8 ^a^
2-undecanone	0.38 ^c^	1.07 ^a^	0.58 ^bc^	0.99 ^ab^	0.67 ^abc^	0.94 ^ab^	0.66 ^abc^	1.00 ^ab^
3-hexen-1-ol	1885 ^c^	2833 ^b^	1837 ^c^	3509 ^a^	1900 ^c^	3222 ^ab^	2055 ^c^	3724 ^a^
3-hexenal	3.21 ^e^	4.40 ^cde^	5.02 ^bcde^	7.01 ^b^	3.74 ^de^	6.31 ^bc^	5.59 ^bcd^	11.8 ^a^
3-methyl-1-butanol	18.1 ^e^	23.3 ^e^	79.9 ^cd^	134 ^b^	52.9 ^de^	152 ^b^	117 ^bc^	276 ^a^
acetic acid	9.12 ^bc^	11.8 ^b^	9.47 ^bc^	12.3 ^b^	4.51 ^d^	9.46 ^bc^	8.48 ^c^	18.4 ^a^
acetoin	3.91 ^de^	4.61 ^cd^	4.34 ^cd^	5.78 ^ab^	3.23 ^e^	4.89 ^bc^	4.27 ^cd^	5.83 ^a^
acetone	12.2 ^e^	17.2 ^d^	24.6 ^c^	31.3 ^b^	15.7 ^de^	29.9 ^b^	29.6 ^b^	52.1 ^a^
alpha-terpinene	1.66 ^c^	1.77 ^c^	1.65 ^c^	2.51 ^ab^	1.90 ^bc^	2.35 ^abc^	2.03 ^bc^	2.79 ^a^
ammonia	536 ^d^	847 ^bc^	692 ^cd^	1262 ^a^	538 ^d^	1008 ^ab^	749 ^cd^	1206 ^a^
beta-caryophyllene	0.90 ^ab^	0.72 ^ab^	0.53 ^b^	1.24 ^a^	0.78 ^ab^	0.51 ^b^	0.45 ^b^	0.53 ^b^
carbon disulfide	888 ^e^	1770 ^cd^	1041 ^e^	2660 ^ab^	988 ^e^	2203 ^bc^	1230 ^de^	2902 ^a^
decanal	1.54 ^c^	1.83 ^bc^	1.67 ^bc^	2.75 ^a^	1.34 ^c^	2.29 ^ab^	1.48 ^c^	2.76 ^a^
dimethyl disulfide	4428 ^c^	8243 ^b^	4565 ^c^	11,437 ^a^	4644 ^c^	10,061 ^ab^	5508 ^c^	12,072 ^a^
dimethyl sulfide	3.59 ^d^	5.62 ^bc^	4.58 ^cd^	6.25 ^b^	3.59 ^d^	6.17 ^b^	5.73 ^bc^	7.92 ^a^
dimethylamine	228 ^b^	525 ^a^	207 ^b^	649 ^a^	213 ^b^	580 ^a^	233 ^b^	685 ^a^
ethyl acetate	4.35 ^de^	5.12 ^cd^	4.82 ^cd^	6.42 ^ab^	3.59 ^e^	5.43 ^bc^	4.74 ^cd^	6.47 ^a^
formaldehyde	1138 ^c^	1433 ^b^	1154 ^c^	1718 ^a^	1148 ^c^	1621 ^ab^	1230 ^c^	1775 ^a^
geosmin	7.99 ^c^	22.3 ^b^	6.79 ^c^	34.9 ^a^	8.88 ^c^	29.9 ^ab^	10.8 ^c^	39.4 ^a^
heptanal	3.72 ^e^	4.02 ^e^	4.91 ^de^	7.96 ^b^	4.25 ^e^	6.53 ^c^	5.71 ^cd^	10.0 ^a^
heptane	224 ^e^	410 ^de^	446 ^de^	1069 ^b^	281 ^e^	710 ^cd^	826 ^bc^	2256 ^a^
hexanal	4.70 ^e^	5.91 ^e^	31.6 ^cd^	41.6 ^bc^	23.2 ^d^	46.3 ^b^	46.3 ^b^	108 ^a^
hexanoic acid	0.85 ^e^	1.22 ^cde^	1.15 ^de^	1.90 ^abc^	1.24 ^cde^	2.18 ^ab^	1.55 ^bcd^	2.53 ^a^
hydrogen sulfide	0.71 ^a^	0.77 ^a^	0.77 ^a^	0.96 ^a^	0.88 ^a^	1.74 ^a^	1.09 ^a^	4.76 ^a^
indole	0.84 ^bc^	1.06 ^bc^	0.64 ^c^	2.29 ^a^	0.65 ^c^	1.58 ^ab^	0.54 ^c^	2.28 ^a^
isobutyl alcohol	7877 ^c^	12,089 ^b^	8126 ^c^	15,687 ^a^	8158 ^c^	14,140 ^ab^	9257 ^c^	16,272 ^a^
methyl mercaptan	8730 ^d^	12,708 ^bc^	9121 ^d^	16,191 ^a^	9106 ^d^	14,665 ^ab^	10,272 ^cd^	16,857 ^a^
nonanal	10.0 ^e^	20.5 ^cd^	12.2 ^de^	32.9 ^ab^	10.8 ^e^	27.3 ^bc^	13.9 ^de^	38.3 ^a^
oct-2-en-1-ol	255 ^c^	756 ^b^	234 ^c^	1175 ^a^	263 c	990 ^ab^	324 ^c^	1270 ^a^
octanal	2.30 ^d^	2.54 ^d^	3.52 ^cd^	4.88 ^b^	2.77 ^d^	4.42 ^bc^	4.27 ^bc^	8.73 ^a^
octane	34.3 ^cd^	54.4 ^bc^	30.3 ^d^	85.0 ^a^	30.9 ^d^	68.8 ^ab^	38.0 ^cd^	90.1 ^a^
pentanal	6.52 ^e^	6.24 ^e^	18.4 ^cd^	28.7 ^bc^	10.6 ^de^	16.8 d	36.0 ^b^	65.4 ^a^
pentane	3002 ^cd^	3781 ^c^	3143 ^cd^	4832 ^b^	1385 ^e^	2917 ^d^	3043 ^cd^	6624 ^a^
propanal	13.6 ^f^	14.5 ^f^	124 ^de^	143 ^cd^	88.3 ^e^	186 ^b^	174 ^bc^	308 ^a^
trans-2-undecenal	5.23 ^c^	10.9 ^bc^	6.63 ^c^	19.4 ^a^	7.04 ^c^	15.6 ^ab^	7.76 ^c^	19.2 ^a^
trimethylamine	20.9 ^e^	27.5 ^e^	64.0 ^c^	77.1 ^bc^	45.2 ^d^	82.8 ^b^	82.8 ^b^	132 ^a^
trimethylpyrazine	11.5 ^c^	29.0 ^b^	12.6 ^c^	42.8 ^a^	13.3 ^c^	36.7 ^ab^	16.5 ^c^	46.8 ^a^

**Table A5:** Effect of sex on the volatile profile of cooked salmon (ppb). Different letters indicate a significant difference in each volatile (*p* < 0.05).

Volatile (ppb)	Female, Raw	Male, Raw	Female, Pan Fried	Male, Pan Fried	Female, Boiled	Male, Boiled	Female, Baked	Male, Baked
(E)-2-pentenal	125 ^bc^	111 ^c^	154 ^ab^	115 ^c^	142 ^abc^	117 ^c^	162 ^a^	128 ^bc^
(E,Z)-2,6-nonadienal	275 ^bcd^	230 ^d^	342 ^ab^	246 ^cd^	318 ^abc^	242 ^cd^	366 ^a^	279 ^abcd^
1-hexanol	3.61 ^d^	3.38 ^d^	6.53 ^c^	6.12 ^c^	7.71 ^bc^	6.17 ^c^	12.5 ^a^	9.30 ^b^
1-octanol	2.77 ^de^	1.97 ^e^	4.70 ^b^	3.31 ^cd^	4.11 ^bc^	2.93 ^de^	6.25 ^a^	4.72 ^b^
1-octen-3-ol	717 ^bcd^	455 ^d^	1107 ^ab^	528 ^cd^	932 ^abc^	522 ^cd^	1191 ^a^	659 ^cd^
1-octen-3-one	0.80 ^bc^	0.60 ^c^	1.17 ^b^	0.86 ^bc^	0.93 ^bc^	1.12 ^b^	1.85 ^a^	1.05 ^bc^
1-pentanol	10.4 ^de^	9.14 ^e^	15.7 ^bc^	15.6 ^bcd^	15.2 ^bcd^	13.5 ^cde^	27.1 ^a^	19.5 ^b^
1-penten-3-ol	11.5 ^d^	10.7 ^d^	42.6 ^c^	44.3 ^c^	44.6 ^c^	37.0 ^c^	90.5 ^a^	64.2 ^b^
2,3-butanediol	68.3 ^bc^	59.1 ^c^	71.6 ^bc^	70.2 ^bc^	39.8 ^d^	29.9 ^d^	95.1 ^a^	76.9 ^b^
2,4-decadienal	0.63 ^de^	0.56 ^e^	1.81 ^bc^	1.58 ^bc^	1.88 ^bc^	1.34 ^cd^	3.17 ^a^	2.27 ^b^
2,4-heptadienal	20.9 ^d^	21.0 ^d^	191 ^c^	206 ^c^	215 ^c^	192 ^c^	400 ^a^	318 ^b^
2-acetyl furan	1.47 ^d^	1.52 ^d^	2.54 ^ab^	1.64 ^cd^	2.32 ^abc^	2.02 ^bcd^	2.93 ^a^	2.30 ^abc^
2-butenal	1.97 ^d^	1.82 ^d^	5.15 ^c^	5.17 ^c^	5.78 ^bc^	4.94 ^c^	9.11 ^a^	6.85 ^b^
2-decanone	0.85 ^b^	1.10 ^b^	1.04 ^b^	0.73 ^b^	1.82 ^a^	1.17 ^ab^	1.19 ^ab^	1.16 ^b^
2-decenal	2.44 ^c^	2.22 ^c^	3.92 ^b^	3.33 ^bc^	3.37 ^bc^	2.93 ^bc^	5.40 ^a^	3.23 ^bc^
2-ethyl-2,5,-dimethylpyrazine	3.38 ^bc^	1.74 ^c^	4.81 ^ab^	2.78 ^c^	3.05 ^bc^	2.57 ^c^	5.48 ^a^	3.32 ^bc^
2-ethylfuran	26.5 ^c^	21.3 ^c^	36.8 ^ab^	25.0 ^c^	31.3 ^abc^	23.7 ^c^	40.2 ^a^	29.2 ^bc^
2-heptanone	5.69 ^ab^	4.90 ^b^	5.52 ^ab^	5.24 ^ab^	6.46 ^a^	6.00 ^ab^	5.80 ^ab^	6.45 ^a^
2-heptenal	12.9 ^cd^	9.72 ^d^	25.0 ^b^	15.9 ^bcd^	20.8 ^bc^	14.1 ^cd^	42.7 ^a^	22.5 ^bc^
2-hexenal	12.2 ^bc^	9.16 ^c^	17.1 ^b^	13.5 ^bc^	15.7 ^b^	11.5 ^bc^	27.4 ^a^	15.6 ^b^
2-isopropyl-3-methoxypyrazine	8.58 ^bc^	5.52 ^c^	14.1 ^b^	9.24 ^bc^	11.1 ^bc^	8.28 ^bc^	21.0 ^a^	12.3 ^b^
2-methyl naphthalene	9.33 ^de^	7.83 ^e^	17.7 ^ab^	11.6 ^cde^	13.1 ^bcd^	9.40 ^de^	20.6 ^a^	15.0 ^bc^
2-methylisoborneol	3.04 ^c^	2.33 ^c^	13.8 ^b^	10.9 ^b^	16.0 ^b^	10.8 ^b^	30.5 ^a^	16.2 ^b^
2-nonanone	1.29 ^a^	0.62 ^b^	1.00 ^ab^	1.12 ^ab^	1.37 ^a^	0.84 ^ab^	1.41 ^a^	1.34 ^a^
2-nonenal	78.5 ^bcd^	48.7 ^d^	123 ^ab^	60.5 ^cd^	99.0 ^abc^	56.2 ^cd^	134 ^a^	78.4 ^bcd^
2-octenal	1.23 ^cd^	1.16 ^d^	2.14 ^b^	1.81 ^bc^	2.01 ^b^	1.55 ^bcd^	2.78 ^a^	2.07 ^b^
2-octene	8.46 ^bc^	6.74 ^c^	12.4 ^ab^	7.50 ^c^	12.0 ^ab^	8.31 ^bc^	13.7 ^a^	9.32 ^abc^
2-pentanone	1.17 ^d^	1.12 ^d^	1.99 ^abc^	1.66 ^cd^	1.90 ^bc^	1.48 ^cd^	2.52 ^a^	2.46 ^ab^
2-penten-1-ol	8.49 ^d^	7.85 ^d^	31.4 ^c^	32.7 ^c^	32.9 ^c^	27.3 ^c^	66.7 ^a^	47.4 ^b^
2-pentene	15.3 ^de^	13.5 ^e^	23.2 ^bc^	23.1 ^bcd^	22.5 ^bcd^	19.9 ^cde^	40.1 ^a^	28.8 ^b^
2-undecanone	1.00 ^a^	0.46 ^b^	0.94 ^a^	0.63 ^ab^	0.86 ^ab^	0.74 ^ab^	0.92 ^a^	0.74 ^ab^
3-hexen-1-ol	2500 ^bc^	2218 ^c^	3040 ^ab^	2306 ^c^	2803 ^abc^	2319 ^c^	3223 ^a^	2555 ^bc^
3-hexenal	4.16 ^cd^	3.46 ^d^	5.47 ^bcd^	6.55 ^b^	5.62 ^bc^	4.43 ^cd^	10.8 ^a^	6.53 ^b^
3-methyl-1-butanol	21.9 ^d^	19.6 ^d^	109 ^c^	105 ^c^	114 ^c^	90.8 ^c^	227 ^a^	166 ^b^
acetic acid	12.1 ^b^	8.81 ^bcd^	11.1 ^b^	10.6 ^bc^	7.82 ^cd^	6.14 ^d^	15.6 ^a^	11.2 ^b^
acetoin	4.45 ^bcd^	4.07 ^cd^	5.71 ^a^	4.40 ^cd^	4.54 ^bc^	3.58 ^d^	5.34 ^ab^	4.75 ^bc^
acetone	15.7 ^e^	13.6 ^e^	27.7 ^c^	28.3 ^c^	25.4 ^c^	20.2 ^d^	43.8 ^a^	37.9 ^b^
alpha-terpinene	1.70 ^b^	1.73 ^b^	2.42 ^ab^	1.74 ^b^	2.19 ^ab^	2.06 ^ab^	2.28 ^ab^	2.53 ^a^
ammonia	763 ^cd^	620 ^d^	1124 ^a^	830 ^cd^	897 ^abc^	649 ^cd^	1102 ^ab^	853 ^bcd^
beta-caryophyllene	0.71 ^a^	0.91 ^a^	0.82 ^a^	0.94 ^a^	0.67 ^a^	0.63 ^a^	0.47 ^a^	0.51 ^a^
carbon disulfide	1461 ^cd^	1198 ^d^	2150 ^ab^	1551 ^bcd^	1834 ^abc^	1358 ^cd^	2404 ^a^	1728 ^bcd^
decanal	1.95 ^bc^	1.43 ^c^	2.67 ^a^	1.75 ^bc^	1.99 ^bc^	1.64 ^bc^	2.27 ^ab^	1.97 ^bc^
dimethyl disulfide	6966 ^bcd^	5705 ^d^	9335 ^ab^	6666 ^cd^	8388 ^abc^	6317 ^cd^	10,128 ^a^	7452 ^bcd^
dimethyl sulfide	5.13 ^bc^	4.08 ^c^	5.69 ^b^	5.14 ^bc^	5.68 ^b^	4.08 ^c^	7.18 ^a^	6.47 ^ab^
dimethylamine	443 ^ab^	310 ^b^	530 ^a^	327 ^b^	480 ^ab^	312 ^b^	563 ^a^	354 ^b^
ethyl acetate	4.95 ^bcd^	4.52 ^cd^	6.35 ^a^	4.89 ^cd^	5.05 ^bc^	3.97 ^d^	5.94 ^ab^	5.28 ^bc^
formaldehyde	1317 ^cd^	1255 ^d^	1530 ^ab^	1342 ^bcd^	1461 ^abc^	1308 ^cd^	1600 ^a^	1404 ^abcd^
geosmin	18.6 ^bcd^	11.7 ^d^	28.0 ^ab^	13.7 ^cd^	24.5 ^abc^	14.3 ^cd^	31.7 ^a^	18.5 ^bcd^
heptanal	3.96 ^de^	3.78 ^e^	7.31 ^b^	5.55 ^c^	5.63 ^c^	5.15 ^cd^	8.57 ^a^	7.17 ^b^
heptane	375 ^e^	259 ^e^	772 ^c^	743 ^cd^	581 ^cde^	410 ^de^	1803 ^a^	1279 ^b^
hexanal	5.55 ^d^	5.06 ^d^	37.9 ^c^	35.2 ^c^	36.9 ^c^	32.6 ^c^	87.4 ^a^	67.1 ^b^
hexanoic acid	1.18 ^bc^	0.89 ^c^	1.44 ^bc^	1.62 ^ab^	1.86 ^ab^	1.56 ^abc^	2.23 ^a^	1.85 ^ab^
hydrogen sulfide	0.77 ^a^	0.71 ^a^	1.06 ^a^	0.67 ^a^	0.65 ^a^	1.97 ^a^	1.28 ^a^	4.57 ^a^
indole	0.87 ^c^	1.02 ^bc^	1.97 ^a^	0.95 ^c^	1.33 ^abc^	0.89 ^c^	1.77 ^ab^	1.05 ^bc^
isobutyl alcohol	10,629 ^bcd^	9337 ^d^	13,287 ^ab^	10,525 ^cd^	12,203 ^abc^	10,096 ^cd^	14,170 ^a^	11,358 ^bcd^
methyl mercaptan	11,276 ^cd^	10,162 ^d^	13,799 ^ab^	11,514 ^bcd^	12,785 ^abc^	10,986 ^cd^	14,804 ^a^	12,324 ^abcd^
nonanal	18.2 ^cd^	12.3 ^d^	27.4 ^ab^	17.7 ^cd^	22.0 ^bc^	16.0 ^cd^	31.3 ^a^	20.8 ^bcd^
oct-2-en-1-ol	618 ^bcd^	393 ^d^	954 ^ab^	455 ^cd^	803 ^abc^	450 ^cd^	1026 ^a^	568 ^cd^
octanal	2.53 ^e^	2.31 ^e^	4.83 ^bc^	3.57 ^cde^	3.93 ^cd^	3.26 ^de^	7.78 ^a^	5.22 ^b^
octane	49.5 ^c^	39.2 ^c^	74.2 ^ab^	41.1 ^c^	57.0 ^abc^	42.6 ^c^	75.3 ^a^	52.7 ^bc^
pentanal	6.78 ^e^	5.98 ^e^	20.2 ^cd^	26.9 ^c^	14.5 ^de^	12.9 ^de^	56.7 ^a^	44.7 ^b^
pentane	3626 ^bc^	3157 ^cd^	4084 ^b^	3891 ^bc^	2404 ^de^	1897 ^e^	5436 ^a^	4231 ^b^
propanal	14.0 ^d^	14.1 ^d^	128 ^c^	139 ^c^	145 ^c^	129 ^c^	269 ^a^	214 ^b^
trans-2-undecenal	9.52 ^cd^	6.58 ^d^	17.1 ^a^	8.97 ^cd^	13.2 ^abc^	9.47 ^cd^	15.7 ^ab^	11.2 ^bcd^
trimethylamine	26.4 ^d^	22.0 ^d^	71.5 ^c^	69.6 ^c^	68.2 ^c^	59.8 ^c^	116 ^a^	99.1 ^b^
trimethylpyrazine	23.6 ^bcd^	16.8 ^d^	34.7 ^ab^	20.7 ^cd^	30.7 ^abc^	19.4 ^cd^	38.3 ^a^	25.0 ^bcd^

**Table A6:** Effect of presence of skin (muscle vs. skin-on muscle) on the volatile profile of cooked salmon (ppb). Different letters indicate a significant difference in each volatile (*p* < 0.05).

Volatile (ppb)	Skin + Muscle, Raw	Muscle, Raw	Skin + Muscle, Fried	Muscle, Fried	Skin + Muscle, Boiled	Muscle, Boiled	Skin + Muscle, Baked	Muscle, Baked
(E)-2-pentenal	133 ^abc^	103 ^c^	144 ^a^	125 ^abc^	146 ^a^	113 ^bc^	143 ^ab^	148 ^a^
(E,Z)-2,6-nonadienal	295 ^ab^	209 ^c^	311 ^ab^	278 ^abc^	320 ^ab^	240 ^bc^	300 ^ab^	344 ^a^
1-hexanol	3.84 ^e^	3.15 ^e^	6.16 ^d^	6.49 ^cd^	6.13 ^d^	7.76 ^c^	9.79 ^b^	12.0 ^a^
1-octanol	2.86 ^de^	1.88 ^e^	3.57 ^cd^	4.44 ^bc^	3.64 ^cd^	3.40 ^d^	5.12 ^ab^	5.86 ^a^
1-octen-3-ol	782 ^abc^	390 ^c^	900 ^ab^	735 ^abc^	935 ^ab^	518 ^bc^	824 ^abc^	1027 ^a^
1-octen-3-one	0.77 ^bc^	0.62 ^c^	1.23 ^ab^	0.80 ^bc^	1.24 ^ab^	0.81 ^bc^	1.37 ^a^	1.53 ^a^
1-pentanol	10.8 ^de^	8.70 ^e^	17.3 ^bc^	14.1 ^cd^	13.9 ^cd^	14.8 ^bcd^	19.8 ^b^	26.9 ^a^
1-penten-3-ol	11.1 ^d^	11.0 ^d^	45.6 ^c^	41.3 ^c^	38.8 ^c^	42.8 ^c^	64.6 ^b^	90.1 ^a^
2,3-butanediol	58.3 ^d^	69.1 ^cd^	74.0 ^bc^	67.8 ^cd^	37.4 ^e^	32.3 ^e^	88.8 ^a^	83.2 ^ab^
2,4-decadienal	0.69 ^d^	0.50 ^d^	1.54 ^bc^	1.85 ^bc^	1.49 ^c^	1.73 ^bc^	2.22 ^b^	3.22 ^a^
2,4-heptadienal	20.4 ^e^	21.5 ^e^	166 ^d^	231 ^c^	157 ^d^	251 ^bc^	300 ^b^	417 ^a^
2-acetyl furan	1.58 ^cd^	1.40 ^d^	2.24 ^abc^	1.94 ^bcd^	2.16 ^abc^	2.18 ^abc^	2.41 ^ab^	2.82 ^a^
2-butenal	2.04 ^d^	1.76 ^d^	5.09 ^c^	5.24 ^c^	5.25 ^c^	5.47 ^c^	6.97 ^b^	9.00 ^a^
2-decanone	1.01 ^bc^	0.93 ^bc^	1.06 ^bc^	0.72 ^c^	1.72 ^a^	1.28 ^abc^	1.40 ^ab^	0.95 ^bc^
2-decenal	2.45 ^bc^	2.21 ^c^	3.52 ^b^	3.73 ^b^	3.71 ^b^	2.59 ^bc^	3.50 ^b^	5.13 ^a^
2-ethyl-2,5,-dimethylpyrazine	3.01 ^ab^	2.11 ^b^	3.90 ^ab^	3.69 ^ab^	3.50 ^ab^	2.13 ^b^	4.16 ^a^	4.65 ^a^
2-ethylfuran	28.7 ^bc^	19.1 ^c^	32.2 ^ab^	29.6 ^ab^	31.9 ^ab^	23.2 ^bc^	30.5 ^ab^	38.8 ^a^
2-heptanone	5.96 ^abc^	4.63 ^d^	4.92 ^cd^	5.84 ^abcd^	6.92 ^a^	5.54 ^bcd^	5.74 ^abcd^	6.51 ^ab^
2-heptenal	13.9 ^cd^	8.65 ^d^	22.1 ^bc^	18.9 ^bcd^	19.5 ^bc^	15.3 ^cd^	26.5 ^b^	38.7 ^a^
2-hexenal	12.7 ^cd^	8.74 ^d^	16.4 ^bc^	14.2 ^bcd^	15.9 ^bc^	11.3 ^cd^	18.9 ^ab^	24.1 ^a^
2-isopropyl-3-methoxypyrazine	8.18 ^bc^	5.92 ^c^	11.7 ^bc^	11.6 ^bc^	11.5 ^bc^	7.98 ^bc^	12.6 ^b^	20.8 ^a^
2-methyl naphthalene	11.7 ^cd^	5.48 ^e^	18.2 ^ab^	11.2 ^cd^	13.7 ^bcd^	8.83 ^de^	20.7 ^a^	14.9 ^bc^
2-methylisoborneol	3.40 ^d^	1.97 ^d^	11.3 ^c^	13.4 ^bc^	12.5 ^bc^	14.3 ^bc^	18.7 ^b^	28.0 ^a^
2-nonanone	1.08 ^ab^	0.83 ^b^	1.17 ^ab^	0.96 ^ab^	1.30 ^ab^	0.91 ^ab^	1.45 ^a^	1.30 ^ab^
2-nonenal	86.8 ^abc^	40.5 ^c^	98.1 ^ab^	85.2 ^abc^	99.5 ^ab^	55.8 ^bc^	92.7 ^ab^	119 ^a^
2-octenal	1.31 ^de^	1.07 ^e^	2.19 ^ab^	1.75 ^bcd^	1.96 ^abc^	1.59 ^cde^	2.52 ^a^	2.33 ^ab^
2-octene	8.81 ^abc^	6.39 ^c^	11.1 ^ab^	8.79 ^abc^	12.4 ^a^	7.91 ^bc^	10.3 ^abc^	12.7 ^a^
2-pentanone	1.03 ^d^	1.26 ^cd^	1.97 ^b^	1.68 ^bc^	1.76 ^bc^	1.61 ^bc^	2.16 ^b^	2.82 ^a^
2-penten-1-ol	8.21 ^d^	8.13 ^d^	33.6 ^c^	30.5 ^c^	28.6 ^c^	31.6 ^c^	47.6 ^b^	66.5 ^a^
2-pentene	16.0 ^de^	12.9 ^e^	25.6 ^bc^	20.8 ^cd^	20.6 ^cd^	21.8 ^bcd^	29.3 ^b^	39.7 ^a^
2-undecanone	0.79 ^ab^	0.66 ^ab^	0.81 ^ab^	0.76 ^ab^	1.07 ^a^	0.53 ^b^	0.89 ^ab^	0.77 ^ab^
3-hexen-1-ol	2634 ^ab^	2084 ^b^	2861 ^a^	2485 ^ab^	2892 ^a^	2231 ^b^	2852 ^a^	2927 ^a^
3-hexenal	4.31 ^cd^	3.30 ^d^	6.06 ^bc^	5.97 ^bc^	6.23 ^bc^	3.81 ^d^	7.67 ^b^	9.69 ^a^
3-methyl-1-butanol	21.3 ^d^	20.2 ^d^	96.9 ^c^	117 ^c^	87.7 ^c^	117 ^c^	156 ^b^	237 ^a^
acetic acid	9.89 ^bc^	11.0 ^ab^	11.1 ^ab^	10.6 ^b^	7.01 ^c^	6.96 ^c^	13.0 ^ab^	13.9 ^a^
acetoin	4.30 ^b^	4.23 ^b^	5.46 ^a^	4.65 ^ab^	4.20 ^b^	3.92 b	4.79 ^ab^	5.30 ^a^
acetone	15.6 ^e^	13.8 ^e^	29.6 ^c^	26.3 ^cd^	23.4 ^d^	22.2 ^d^	34.6 ^b^	47.1 ^a^
alpha-terpinene	1.67 ^b^	1.76 ^ab^	2.19 ^ab^	1.97 ^ab^	2.10 ^ab^	2.15 ^ab^	2.36 ^ab^	2.46 ^a^
ammonia	856 ^ab^	527 ^c^	1042 ^a^	912 ^a^	900 ^a^	646 ^bc^	981 ^a^	974 ^a^
beta-caryophyllene	0.91 ^ab^	0.70 ^ab^	1.01 ^a^	0.76 ^ab^	0.54 ^ab^	0.76 ^ab^	0.55 ^ab^	0.43 ^b^
carbon disulfide	1642 ^b^	1016 ^c^	1879 ^ab^	1822 ^ab^	1845 ^ab^	1346 ^bc^	1777 ^ab^	2356 ^a^
decanal	2.05 ^abc^	1.32 ^d^	2.54 ^a^	1.88 ^bcd^	2.13 ^ab^	1.50 ^cd^	2.23 ^ab^	2.01 ^abc^
dimethyl disulfide	7688 ^ab^	4982 ^c^	8346 ^ab^	7655 ^ab^	8566 ^ab^	6139 ^bc^	7694 ^ab^	9886 ^a^
dimethyl sulfide	4.95 ^cd^	4.26 ^d^	5.77 ^bc^	5.06 ^cd^	5.38 ^bcd^	4.38 ^d^	6.42 ^ab^	7.23 ^a^
dimethylamine	444 ^ab^	309 ^b^	460 ^ab^	397 ^ab^	469 ^ab^	324 ^ab^	427 ^ab^	491 ^a^
ethyl acetate	4.77 ^b^	4.70 ^b^	6.07 ^a^	5.17 ^ab^	4.67 ^b^	4.35 ^b^	5.32 a^b^	5.89 ^a^
formaldehyde	1400 ^bc^	1172 ^d^	1456 ^abc^	1416 ^bc^	1482 ^ab^	1287 ^cd^	1386 ^bc^	1619 ^a^
geosmin	19.0 ^abc^	11.3 ^c^	22.5 ^ab^	19.2 ^abc^	24.3 ^ab^	14.5 ^bc^	21.4 ^abc^	28.8 ^a^
heptanal	4.69 ^d^	3.05 ^e^	6.90 ^bc^	5.96 ^c^	6.07 ^c^	4.72 ^d^	8.28 ^a^	7.46 ^ab^
heptane	385 ^de^	249 ^e^	856 ^c^	659 ^cd^	563 ^cde^	428 ^de^	1274 ^b^	1807 ^a^
hexanal	5.47 ^d^	5.13 ^d^	32.0 ^c^	41.2 ^c^	30.0 ^c^	39.5 ^c^	68.3 ^b^	86.1 ^a^
hexanoic acid	1.21 ^bc^	0.86 ^c^	1.70 ^b^	1.36 ^bc^	1.64 ^b^	1.78 ^ab^	1.68 ^b^	2.39 ^a^
hydrogen sulfide	0.88 ^ab^	0.60 ^b^	1.02 ^ab^	0.71 ^ab^	0.84 ^ab^	1.77 ^ab^	0.97 ^ab^	4.88 ^a^
indole	0.95 ^a^	0.94 ^a^	1.44 ^a^	1.48 ^a^	1.08 ^a^	1.14 ^a^	1.19 ^a^	1.63 ^a^
isobutyl alcohol	11,499 ^abc^	8467 ^d^	12,256 ^abc^	11,556 ^abc^	12,454 ^ab^	9844 ^cd^	11,440 ^bc^	14,089 ^a^
methyl mercaptan	12,174 ^b^	9264 ^c^	12,933 ^ab^	12,380 ^b^	13,065 ^ab^	10,706 ^bc^	12,120 ^b^	15,009 ^a^
nonanal	20.3 ^ab^	10.2 ^c^	24.9 ^a^	20.2 ^ab^	24.5 ^a^	13.5 ^bc^	26.2 ^a^	26.0 ^a^
oct-2-en-1-ol	674 ^abc^	337 ^c^	776 ^ab^	634 ^abc^	806 ^ab^	447 ^bc^	710 ^abc^	885 ^a^
octanal	2.63 ^d^	2.22 ^d^	4.26 ^c^	4.15 ^c^	3.92 ^c^	3.27 ^cd^	7.22 ^a^	5.78 ^b^
octane	55.2 ^ab^	33.6 ^c^	62.4 ^ab^	52.9 ^abc^	55.1 ^ab^	44.5 ^bc^	58.4 ^ab^	69.6 ^a^
pentanal	4.83 ^f^	7.93 ^ef^	28.2 ^c^	18.9 ^cd^	12.2 ^def^	15.3 ^de^	45.3 ^b^	56.1 ^a^
pentane	3405 ^c^	3378 ^c^	4060 ^bc^	3915 ^bc^	2351 ^d^	1950 ^d^	4604 ^ab^	5063 ^a^
propanal	13.7 ^e^	14.4 ^e^	111 ^d^	155 ^c^	105 ^d^	168 ^bc^	202 ^b^	281 ^a^
trans-2-undecenal	9.47 ^bc^	6.63 ^c^	12.1 ^abc^	13.9 ^ab^	11.8 ^abc^	10.9 ^abc^	11.2 ^abc^	15.7 ^a^
trimethylamine	24.7 ^e^	23.7 ^e^	66.9 ^cd^	74.2 ^c^	55.4 ^d^	72.6 ^c^	91.2 ^b^	124 ^a^
trimethylpyrazine	25.9 ^ab^	14.5 ^c^	28.5 ^ab^	26.9 ^ab^	30.3 ^ab^	19.7 ^bc^	26.4 ^ab^	36.9 ^a^
